# Impact of 14 days of head‐down bed rest and an exercise countermeasure on skeletal muscle atrophy, proteome and circulatory cytokines in older adults

**DOI:** 10.1113/EP093524

**Published:** 2026-03-27

**Authors:** Jean‐Christophe Lagacé, Philippe St‐Martin, Mariano Avino, Ahmed Ghachem, Guy Hajj‐Boutros, Jose A. Morais, Jamie S. McPhee, Karen Lambert, Eléonor Riesco, Isabelle J. Dionne

**Affiliations:** ^1^ Faculty of Physical Activity Sciences Université de Sherbrooke Sherbrooke Quebec Canada; ^2^ Research Centre on Aging CIUSSS de l'Estrie – CHUS Sherbrooke Quebec Canada; ^3^ Centre National de la Recherche Scientifique, Institut Pluridisciplinaire Hubert Curien UMR 7178 Université de Strasbourg Strasbourg France; ^4^ Bio‐informatic Platform, Department of Biochemistry and Functional Genomics Faculty of Medicine and Health Sciences Université de Sherbrooke Sherbrooke Quebec Canada; ^5^ Research Institute of McGill University Health Centre McGill University Montréal Quebec Canada; ^6^ Department of Sport and Exercise Sciences Manchester Metropolitan University Manchester UK; ^7^ PhyMedExp Université de Montpellier, INSERM, CNRS Montpellier France

**Keywords:** ageing, bed rest, exercise, proteomics, skeletal muscle

## Abstract

Prolonged bed rest and immobilization have deleterious effects on skeletal muscle mass and function, especially in older adults. These can lead to reduced physical capacity and quality of life. Previous experiments in younger individuals show that exercise can mitigate these effects, but evidence remains scarce and inconclusive in older adults. Therefore, the aim of this study was to evaluate the effects of a mixed‐exercise countermeasure on body composition, muscle volume, strength and quality in healthy, ageing adults during 2 weeks of head‐down tilt bed rest and in the subsequent recovery period, and to explore the underlying mechanisms. Twenty‐two participants (11 male and 11 female) aged 55–65 years completed the head‐down tilt bed rest protocol. Participants were randomly assigned to either an exercise group (*n* = 11) undertaking three daily exercise sessions or a passive‐mobilization control group (*n* = 11). Assessments of body composition, thigh muscle volume, muscle strength, blood markers and muscle biopsies were performed before, during and after bed rest, and after 4 weeks and 4 months of recovery. Bed rest induced significant declines in total and lower‐limb lean body mass, knee‐extensor strength and muscle quality. The mixed‐exercise intervention mitigated the loss of thigh muscle volume but did not prevent reductions in total or lower‐limb lean mass, strength or muscle quality. All participants had recovered fully after 4 weeks of recovery. In conclusion, daily participation in an exercise countermeasure preserves thigh muscle volume during 2 weeks of bed rest in healthy older adults but is insufficient to mitigate muscle strength and total lean mass.

## Abstract (highlights)

- **What is the central question of this study?**
  How does a mixed‐exercise countermeasure affect muscle atrophy, strength and subsequent recovery after 14 days of head‐down bed rest?
- **What is the main finding and its importance?**
  The mixed‐exercise intervention mitigated the loss of thigh muscle volume but did not prevent reductions in total or lower‐limb lean mass, strength or muscle quality. All participants had recovered fully after 4 weeks.

## 1 INTRODUCTION

Muscle disuse is commonly observed during prolonged bed rest and immobilization and confers deleterious effects on muscle mass and function (Di Girolamo et al., 2021; Dirks et al., 2016; Kehler et al., 2019; Kortebein et al., 2008; Marusic et al., 2021; Pisot et al., 2016; Reidy et al., 2018; Rudrappa et al., 2016; Tanner et al., 2015; Wall & van Loon, 2013; Wall et al., 2014). These deleterious effects were shown to occur in a progressive, yet non‐linear fashion, with muscle atrophy and loss of strength occurring in the earlier stages of bed rest (5–14 days) and reaching a plateau after 1 month (Marusic et al., 2021). Muscle atrophy and loss of strength are especially prevalent in lower extremities (Di Girolamo et al., 2021; Kehler et al., 2019), which can impair physical function and ultimately lead to loss of functional capacity in older individuals (Coker et al., 2015). Indeed, a meta‐analysis found that a 10 day period of bed rest resulted in decreased knee‐extension power, stair‐climbing power, maximal voluntary isometric force, walking speed and chair‐stand time and that these effects were exacerbated in older individuals compared with younger counterparts (Di Girolamo et al., 2021). Furthermore, several studies have shown that older adults experience a greater relative loss of leg lean mass following 5, 7, 10 and 14 days of bed rest (Pisot et al., 2016; Reidy et al., 2018; Tanner et al., 2015). These findings indicate that older adults are particularly vulnerable to the deleterious effects of prolonged immobilization, yet studies investigating the effect of exercise countermeasures to attenuate muscle and strength loss remain very limited.

Although previous research has demonstrated that exercise can effectively counteract the detrimental effects of bed rest on muscle atrophy and function in younger adults (Konda et al., 2019; Lee et al., 2014), there is a significant lack of research investigating the effects of exercise countermeasures in bedridden older adults. To our knowledge, the sole study examining such an intervention in older individuals undergoing 7 days of horizontal bed rest reported no improvement in aerobic capacity, muscle strength and muscle fibre volume following a 2000 steps/day protocol (Arentson‐Lantz et al., 2019), probably owing to the relatively low intensity of the intervention. Likewise, previous studies have reported that 2 weeks of multimodal exercise interventions initiated after 14 days of bed rest were insufficient to restore quadriceps muscle volume and lower‐leg muscle power fully to baseline levels in older adults (Pisot et al., 2016; Rejc et al., 2018). Hence, it would be important to investigate whether initiating the higher‐intensity multimodal countermeasure during bed rest, rather than after, could prevent some of the deleterious effects of immobilization and improve the subsequent recovery.

Considering these research gaps, the objectives of the present study were as follows: (1) to evaluate the effects of a mixed‐exercise countermeasure on body composition, muscle volume strength and quality in healthy ageing adults during 2 weeks of head‐down tilt bed rest and in the subsequent recovery period; and (2) to explore associated changes in muscle proteomics and inflammatory markers to gain insight into the mechanisms underlying the observed physiological adaptations.

## 2 MATERIALS AND METHODS

### 2.1 Ethical approval

All participants gave their written informed consent before participating in the study. The study conformed to the *Declaration of Helsinki* and was approved by the research ethics committee of the host institution, McGill University Health Center (MP‐37‐2021‐7170), the local research ethics committee (CIUSSS de l'Estrie—CHUS; #2021‐7170) and other satellite sites.

This study is part of a broader pan‐Canadian study, the Bed Rest in Older Adults (BROA) study, which had a dual objective. First, it aimed to investigate whether exercise can counteract the negative effects of a 2 week head‐down bed rest on muscle function and metabolism, postural control, bone structure, orthostatic tolerance and cognitive function in adults (Clinicaltrial.gov identifier: NCT04964999). This study used the −6° head‐down bed rest model as a microgravity analogue to extend the outcomes to the astronaut population, because the Canadian Space Agency was a co‐sponsor of the project. This model is widely recognized as the most accessible ground‐based analogue to microgravity and has been used in multiple studies (Pavy‐Le Traon et al., 2007). Furthermore, given the nature of the project, where eight different teams and the Canadian Space Agency were involved and shared variables, some of the data have previously been published elsewhere and are reported herein to give better context to the reader. Notably, pre and post bed rest body composition by dual energy X‐ray absorptiometry (DXA) and isometric knee extension have been published by Hajj‐Boutros et al. (2023), and pre and post bed rest thigh muscle volumes were reported by Dulac et al. (2025). However, a more nuanced interpretation of the changes in body composition is offered in this manuscript compared with the previous publication (Hajj‐Boutros et al., 2023). Data for handgrip strength, muscle quality, muscle fat content, proteomic and plasma inflammatory markers, in addition to all data from the 4‐week and 4‐month follow‐ups, are unique to this manuscript.

### 2.2 Population

**FIGURE 1:**
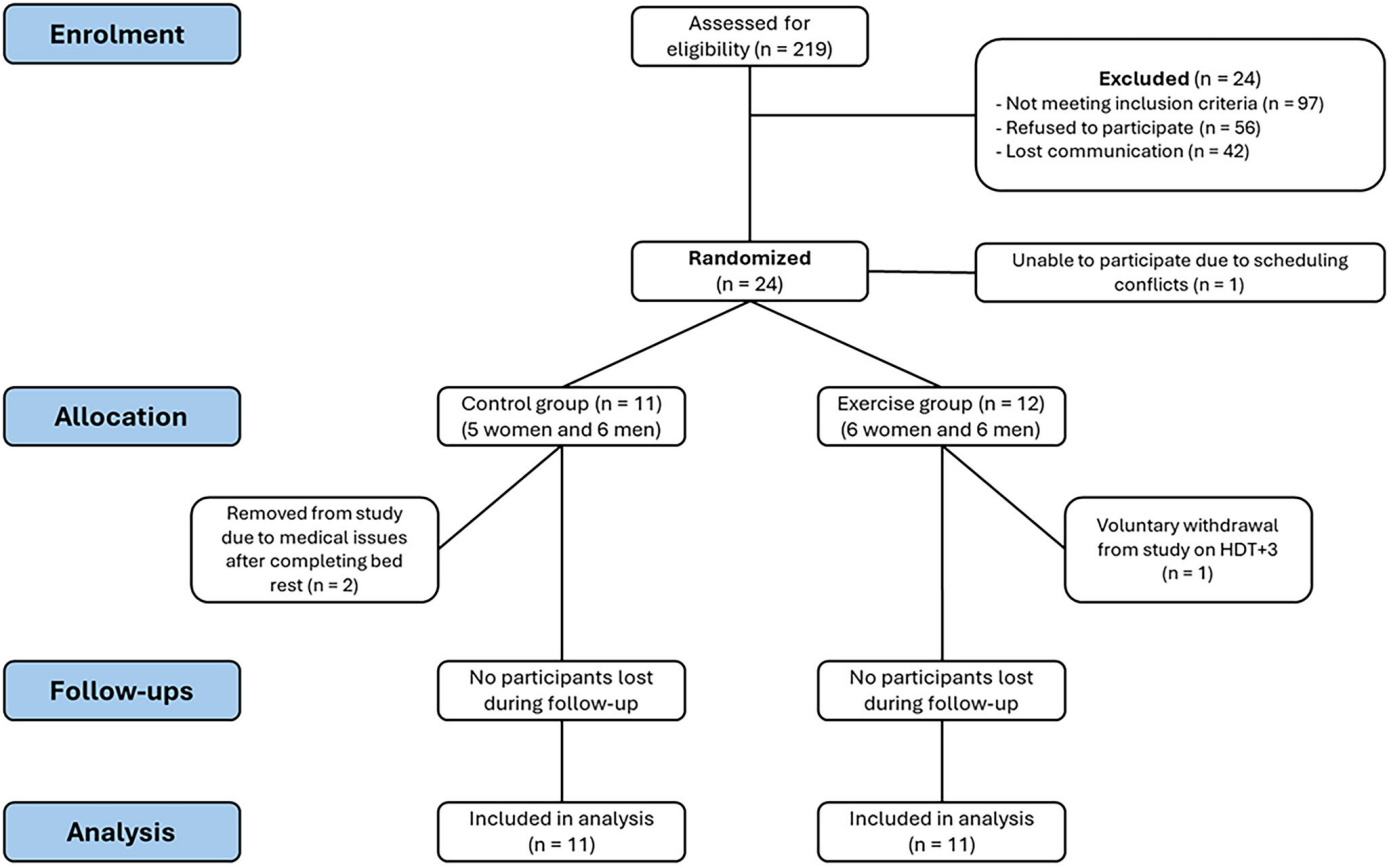
Flow chart of participant enrolment.

A total of 23 participants (11 female and 12 male) took part in the study, with 22 completing the bed rest and 20 completing the full protocol (Figure 1). One participant from the exercise group ended their participation prematurely owing to difficulty in complying with the bed rest protocol, and two participants from the control group were withdrawn on the third day of the recovery period, after completing the bed rest, owing to incidental findings described elsewhere (Hajj‐Boutros et al., 2023). Given that they had completed the entire bed rest period and most of the post bed rest assessments, data from these two participants were included in the analyses up to the point of their withdrawal.

In brief, participants were eligible for inclusion if they were between 55 and 65 years of age (women were amenorrhoeic for >1 year), measured between 158 and 195 cm in height, and had a body mass index between 20 and 35 kg/m^2^. All participants were required to be physically healthy, as confirmed by an extensive medical screening, and mentally healthy, as determined by successful completion of the Positive and Negative Affect Schedule and the General Health Questionnaires. A detailed description of the inclusion and exclusion criteria has been published previously (Hajj‐Boutros et al., 2024).

### 2.3 Protocol overview

**FIGURE 2:**
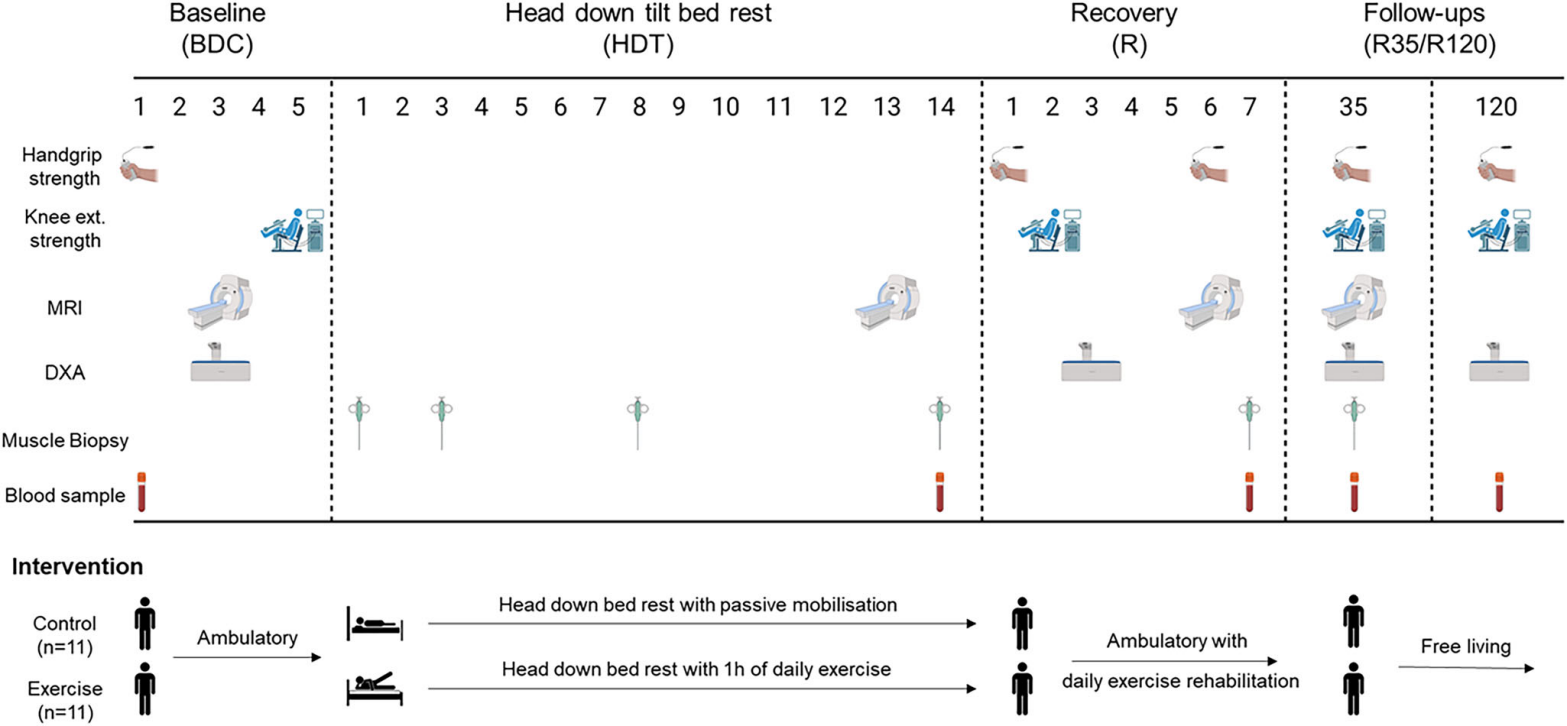
Study design.

The methodology reported here represents the specific approach used for the results reported. The reader is referred to the full protocol previously published (Hajj‐Boutros et al., 2024). In brief, following an initial visit to assess eligibility, participants returned to the research facility the evening before the beginning of the second visit to facilitate adaptation to the study environment. Participants remained in the facility for 26 consecutive days (Figure 2), consisting of 5 days of baseline data collection (BDC1–BDC5), 14 days of −6° head‐down tilt (HDT1–HDT14) and 7 days of recovery (R1–R7). Participants were ambulatory throughout the baseline and recovery periods and remained in a −6° head‐down position during the bed rest period (HDT1–HDT14). The research staff attended to all participants’ needs for the duration of their stay. Finally, participants came back for two follow‐up visits 4 weeks (R35) and 4 months (R120) after discharge.

### 2.4 Exercise intervention

The standardized exercise countermeasure protocol was developed by the Canadian Space Agency (study sponsor) and adapted by a trained kinesiologist from the McGill University Health Centre (MUHC). The intervention comprised three individually supervised exercise sessions per day, each separated by ∼4 h, for a total of 60–75 min of physical exercise daily. Exercises were performed in a dedicated room adjacent to the facility, and participants remained in a head‐down tilt (HDT) or horizontal position, depending on the specific movement and available equipment, in order to replicate microgravity conditions.

Detailed information regarding the distribution of training sessions during bed rest, in addition to the intensity and movements can be found in Appendix 1. Briefly, sessions consisted in either aerobic exercise on a cycle ergometer (Ergoselect 8, Ergoline GmbH, Germany) or resistance training targeting upper‐ or lower‐body muscle groups using body weight, exercise bands and a cable/pulley station. There were three types of aerobic sessions: continuous (long or short sessions), progressive aerobics (pyramid‐type sessions) and high‐intensity interval training sessions. The exercise intensity during aerobic sessions was set as a percentage of heart rate reserve. Resistance exercise sessions comprised three sets, including one set of warm‐up, of 10–12 repetitions. No prior maximal testing was completed, and the intensity and weight were adjusted based on the judgement of the supervising kinesiologist.

### 2.5 Diet

A weight‐maintaining diet based on caloric needs and weight fluctuations (daily weighing) of participants was prepared by the study nutritionists throughout the 26 day inpatient period. Resting caloric needs were assessed with indirect calorimetry, and multiplicative activity factors of 1.5 and 1.3, including the thermic effect of food, were used to determine energy requirements during ambulatory and bed rest periods, respectively. The exercise group maintained the 1.5 activity factor to account for energy requirements of exercise. The standardized diet consisted of ∼50% carbohydrates, 35% fats and, based on recent expert recommendations for protein intake in older adults (Bauer et al., 2013), the minimal targeted protein intake was 1.2 g/kg/day. Further details of the diet were published elsewhere (Guan, 2022).

### 2.6 Blood samples and biochemistry

Blood samples were collected after an overnight fast at baseline (BDC1), on the last morning of bed rest (HDT14), at the end of the recovery period (R7) and during both follow‐up visits at 4 weeks (R35) and 4 months (R120). Samples were immediately placed on ice and centrifugated within 15 min of collection. Centrifugation was performed for 10 min at 1900*g* and 4°C. The resulting plasma was aliquoted and stored at −80°C until further analysis.

Plasma concentrations of tumour necrosis factor‐α (TNF‐α), interleukin‐6 (IL‐6) and interleukin‐8 (IL‐8) were measured using a multiplex enzyme‐linked immunoassay (HSTCMAG‐28SK, Millipore‐Sigma, MA, USA). To minimize technical variability, all samples from a given participant were analysed on the same assay plate. Participants were processed in order of study completion, irrespective of group allocation or sex. All samples were assayed in duplicate, and samples with a coefficient of variation of >15% were excluded from the analysis. Low‐ and mid‐concentration quality control samples were included on each plate to assess intra‐ and interassay consistency. Assay performance met manufacturer‐recommended criteria for reliability. The intra‐assay coefficient of variation for quality control samples ranged from 0.07% to 14.2%, and the interassay coefficient of variation ranged from 2.6% to 17.8%, remaining below the respective 15% and 20% thresholds recommended by the manufacturer. Values below the limit of detection (TNF‐α, 0.16 pg/mL; IL‐6, 0.11 pg/mL; and IL‐8, 0.13 pg/mL) were treated as missing.

### 2.7 Muscle strength

Strength was assessed in both the upper and lower limbs at baseline, after bed rest, after recovery and during follow‐up visits. However, to avoid interfering with the study protocol, strength assessments were not performed during the bed rest period.

Handgrip strength was measured using a Jamar hand‐held dynamometer following standard procedures. Briefly, participants stood upright with their arm fully extended but not in contact with the body. The dynamometer was adjusted to fit comfortably in each participant's hand. Participants were instructed to squeeze the dynamometer as forcefully as possible for ∼3 s, while maintaining their position. The test was performed twice with each hand, alternating hands between efforts. The sum of the highest value from each hand was used as the handgrip strength measure.

A quantitative multi‐joint Biodex dynamometer (Biodex System 3, Mirion Technologies Inc., NJ, USA) was used to assess maximal voluntary strength of the knee‐extensor muscles on the right leg. After a brief warm‐up, participants were positioned on the Biodex and secured with belts to minimize extraneous movement. Standardized verbal instructions were provided, and the exact positioning was recorded to ensure replicability. Each participant performed three isometric contractions at a knee and hip angle of 90°, each lasting 5 s, with 60 s of rest between contractions. Standardized verbal encouragements were provided throughout the test. The maximal torque value from obtained the three contractions was considered as maximal voluntary strength.

### 2.8 Body composition

Body composition was determined using a DXA scan (Lunar Progidy DXA, General Electric Healthcare, USA), which accurately estimates bone mineral content, lean body mass (LBM) and fat mass (FM). Body composition was measured at baseline (BDC3), after bed rest (R3) and during both follow‐up visits (R35 and R120). The different regions of interest (upper body, lower body and trunk) were defined by a trained investigator. The consensus for the coefficient of variation for lean mass and fat mass using the DXA method is ∼2% and ∼3%, respectively (Hangartner et al., 2013).

### 2.9 Thigh muscle and fat volume

Thigh muscle volume and fat infiltration were determined using a 3 T MRI (MR750 scanner, GE Healthcare, Chicago, IL, USA; GE 36 channel peripheral coil) at multiple time points: BDC1, HDT13, R6 and R35. Details of the image acquisition and image post‐processing were published elsewhere (Dulac et al., 2025). Briefly, scans were conducted at the same time of day for each participant, who refrained from exercise or strenuous activity for 10 h prior to scanning. To minimize the influence of fluid shifts on muscle volume, participants rested in a supine horizontal position for 1 h prior to scanning, following the protocol described by Berg et al. (1993). Participants were positioned feet‐first in the scanner, with a compression device placed between their legs and the scanner table to ensure stability.

An Ideal IQ sequence was used to acquire images of the right thigh muscles. Images were obtained with a targeted resolution of 1 mm × 1 mm × 10 mm, a matrix size of 256 × 256 voxels and a field of view measuring 300 mm × 300 mm. Six echoes were used, with a repetition time of 10 ms. The acquired MR images were analysed using 3D Slicer software (Fedorov et al., 2012; https://www.slicer.org). The cross‐sectional area of the thigh muscles was traced twice, on two different and consecutive images, and the average measurement was used for the calculation of muscle volume, fat volume, intermuscular adipose tissue volume and intramyocellular volume. Intermuscular adipose tissue and intramyocellular lipid volumes were determined by automated volumetric analysis using 3D Slicer, based on the manually segmented regions of interest. All measurements were performed by the same investigator to ensure consistency and minimize variability.

### 2.10 Muscle quality

Muscle quality was calculated as the ratio of maximal isometric knee‐extensor force (in newton metres) to thigh muscle volume (in centimetres cubed; measured with MRI). Given that strength and imaging assessments were performed on separate days according to the study schedule, different time points were used to derive muscle quality. Baseline muscle quality combined strength data from BDC5 with muscle volume from BDC3. Post‐bed rest muscle quality was calculated using strength measured at R2 and muscle volume from HDT13. Follow‐up measurements for both variables were obtained on R35.

### 2.11 Muscle biopsies

Skeletal muscle biopsies were performed using the Bergström technique, as previously described (Dulac et al., 2025). Briefly, a skin portion of the vastus lateralis was prepared and anaesthetized with lignocaine (2.5 mL, 2%). A small incision (6 mm) was then made in the skin and facia with a sterilized scalpel. A UHC needle (5 mm in diameter) was inserted 5–7 cm deep into the vastus lateralis, and suction was used to facilitate sampling. Multiple (two or three) samplings were performed in the same pass by rotating the needle to increase the volume of samples obtained to ∼150–200 mg. Steri‐strips were used to close the incision, and a compression bandage was applied. A total of six biopsies were performed throughout the study (Figure 2). The biopsies were stored at −80°C within 10–15 min of sample collection. The biopsies were executed alternately on each of the two legs, and a gap of 3–4 cm was maintained between biopsies performed on a single leg in accordance with recent recommendations (Long et al., 2023). Although no studies specific to the proteome could be identified, it has been shown previously that linear serial biopsies taken 2.5 cm distal to each other on the vastus lateralis did not affect the muscle transcriptome (Murton et al., 2013).

### 2.12 Peptide purification

Approximately 20 mg of frozen skeletal muscle tissue was placed in 500 µL of lysis buffer (8 M urea, 1 M ammonium bicarbonate and 20 mM Hepes KOH, pH 7.5). A 5 mm stainless‐steel bead was added to the solution, and the tissue was homogenized mechanically for 3 × 2 min at 50 oscillations/s in a cooled adapter (TissueLyser LT, Quiagen, Germany) with a 2 min rest on ice in between each passage. The homogenate was centrifuged at 21 100*g* for 5 min at 4°C, and the supernatant was collected. Proteins were then quantified using a Pierce BCA protein assay (ThermoFisher Scientific, MA, USA). A total of 500 µg of proteins was transferred in a low‐bind aliquot for digestion, and a lysis buffer was added to obtain 500 µL (8 M urea, 1 M ammonium bicarbonate and 20 mM Hepes KOH, pH 7.5). Dithiothreitol (5 mM) was added. The sample was boiled for 2 min at 95°C, then incubated for 30 min at room temperature. Chloroacetamide (7.5 mM) was added, and the sample was incubated for another 20 min at room temperature covered with aluminium foil. Ammonium bicarbonate (1 M) was added to dilute to 2 M urea, and 10 µg of trypsin was added. The sample was incubated at 30°C overnight, while rotating at 650 rpm.

The sample was acidified the next morning with trifluoroacetic acid (TFA) to a final concentration of 0.2% TFA. The peptides were purified using 1 mL Zip Tips containing a C18 cartridge (ThermoFisher Scientific, MA, USA). The Zip tip was moistened with three passages of 1 mL acetonitrile 100% and equilibrated with three passages of 1 mL 0.1% TFA before the sample was passed three times. The Zip tip was then washed with three passages of 1 mL 0.1% TFA, and peptides were eluted with three passages of 0.5 mL of elution buffer (50% acetonitrile and 1% formic acid). The peptides were collected in a new low‐bind tube for a final volume of 1.5 mL. They were then concentrated with a centrifugal evaporator (Vacufuge plus, Eppendorf, Germany) at 60°C until completely dry, then resuspended in 1300 µL of 1% formic acid. The peptides were assayed using the NanoDrop 2000 spectrophotometer (Thermo Fisher Scientific, MA, USA) and read at an absorbance of 205 nm. Finally, 20 µL of the sample were transferred into a glass vial (Thermo Fisher Scientific, MA, USA) and stored at −20°C until analysis. Peptides were quantified using liquid chromatography and tandem mass spectrometry (nanoElute, Bruker Daltonics, USA) and subsequently analysed using the DIA‐nn software (Demichev et al., 2020) by the Mass Spectrometry Proteomic Platform at the University of Sherbrooke.

### 2.13 Statistical analysis

The normality of distribution was verified using Shapiro–Wilk tests. Between‐group differences were analysed with Student's unpaired *t*‐tests for normally distributed data (age, weight and body mass index) and with the Mann–Whitney *U*‐test for non‐normally distributed data (height). The main effect of time, group and their interaction were evaluated using linear mixed‐effect models. Sphericity was verified using the Geisser–Greenhouse epsilon, and corrections were applied when required. *Post hoc* contrasts for time were conducted when main effects of time were identified in the models. Baseline values were used as references, and all contrasts were compared against these baseline measures. Multiple comparisons were adjusted using Dunnett's correction. Relationships between changes in knee‐extensor strength (KES) and changes in muscle volume were assessed using Spearman's rank correlations. Results are reported as the median [IQR] unless otherwise specified. The significance level was set at α = 0.05. All statistical analyses were performed using GraphPad Prism v.10.4.1 (Dotmatics, Boston, MA, USA).

### 2.14 Proteomic data processing and analysis

The *proteinGroup.txt* output file generated from the MaxQuant analysis was used to extract corrected reporter intensities per sample for each tandem mass tag mass spectrometryTMT‐MS‐detected protein as a quantitative measure. Given that samples from different time points were run on different days, we assessed potential batch effects by generating a multidimensional scaling plot with the limma package (v.3.65.1) in R. A batch effect was confirmed in the HDT14 samples (data not shown) and was corrected using the internal reference scaling methodology (Plubell et al., 2017), which accounts for random MS2 sampling variability across TMT experiments. For consistency, analyses were performed with and without HDT14; the main results are reported herein, while analyses for HDT14 are presented in the (Figure S10).

Proteins that were not quantified in three participants or more per group were excluded from analyses. Missing data patterns were examined and treated with the DEP2 (v.0.5.28.2) package (Feng et al., 2023). The heatmap was visually inspected (data not shown), and given that missing values were determined to occur at random, they were imputed using random forest‐based imputation (*ntree* = 50; *mtry* = 5).

Differential expression (DE) analysis was performed in R with limma, using a false discovery rate (Benjamini–Hochberg correction) threshold of 0.05 and a log_2_ fold‐change (log_2_ FC) cut‐off of ±0.5. Contrasts were tested within group, using HDT1 (baseline) as a reference. Between‐group contrasts were also performed at each time point. Functional enrichment was assessed through gene ontology (GO) analyses using DEP2 (false discovery rate = 0.05; no log_2_ FC threshold applied). In addition, network analysis and expression pattern clustering were performed with DEP2 to explore biological processes and pathway‐level changes.

## 3 RESULTS

### 3.1 Study sample characteristics

A total of 22 healthy adults completed the bed rest period and were included in the analyses. The descriptive characteristics of participants can be found in Table 1. There were no between‐group differences for age, height, weight or body mass index at baseline (all *P*‐values ≥ 0.176), and the groups were balanced for sex (χ^2 ^= 0.182, *P* = 0.670).

**TABLE 1:** Population characteristics at baseline.

Characteristic	Control (*n* = 11)	Exercise (*n* = 11)
Age, years	58 [56, 61]	59 [57, 61]
Sex, female/male	5/6	6/5
Height, cm	164.7 [160.1, 167.9]	167.2 [159.3, 174.3]
Weight, kg	69.6 [55.5, 75.7]	74.1 [56.8, 87.3]
Body mass index, kg/m^2^	25.0 [21.6, 26.0]	26.3 [22.2, 27.4]

*Note*: Data are presented as the median [interquartile range]. There were no significant differences between groups with *t*‐tests.

### 3.2 Body composition

#### 3.2.1 Lean body mass

**FIGURE 3:**
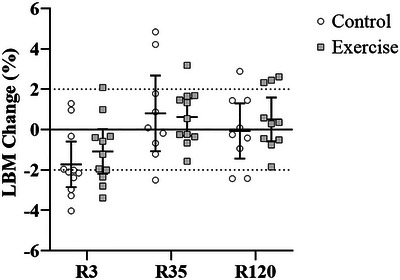
Average changes in LBM compared with baseline. Average proportional changes (as a percentage) of LBM compared with baseline in the control (*n* = 11, white circles) and exercise (*n* = 11; grey squares) groups. Data are presented as the mean change ± 95% confidence interval. The dotted line represents the 2% threshold for test–retest reliability of the dual energy X‐ray absorptiometry for the measurement of LBM. Abbreviation: LBM, lean body mass.

Total LBM (in kilograms) and FM (in kilograms and as a percentage) as well as lower‐limb LBM (in kilograms) and FM (in kilograms) were measured throughout the study using DXA and the median [interquartile range] values are displayed in Table 2. There was a main effect of time for absolute total [*F*(1.152, 21.13) = 10.29, *P* = 0.0031, *n* = 22] and lower‐limb LBM [*F*(1.088, 19.95) = 12.97, *P* = 0.0014, *n* = 22], without group or interaction effects (all *P*‐values *≥* 0.619). *Post hoc* analyses showed that total LBM was reduced after bed rest (R3) compared with baseline [BDC1; mean difference −0.68 kg, 95% confidence interval (CI; −1.12, −0.24), *P* = 0.021, *n* = 22]. Lower‐limb LBM was also reduced after bed rest compared with baseline [mean difference −0.34 kg, 95% CI (−0.58, −0.10), *P* = 0.005, *n* = 22] but was greater at the 4‐week follow‐up (R35) compared with baseline [mean difference 0.29 kg, 95% CI (0.03, 0.55), *P* = 0.026, *n* = 20]. The average relative change in total and lower‐limb LBM was −1.5% [95% CI (−2.4, −0.5)] and −2.0% [95% CI (−3.4, −0.6)], respectively (Figure 3). Neither total nor lower‐limb LBM was different from baseline values at the 4‐month follow‐up (R120; all *P*‐values ≥ 0.157).

**TABLE 2:** Body composition.

Variable	Control				Exercise			
	BDC3 (*n* = 11)	R3 (*n* = 11)	R35 (*n* = 9)	R120 (*n* = 9)	BDC3 (*n* = 11)	R3 (*n* = 11)	R35 (*n* = 11)	R120 (*n* = 10)
LBM, kg^a^	42.9 [38.8,53.5]	43.2 [38.7, 52.4]	41.7 [38.3, 51.8]	42.5 [38.3, 51.0]	41.0 [39.0, 61.1]	39.9 [39.4, 59.7]	40.8 [38.8, 62.1]	47.2 [37.8, 62.3]
FM, kg^b^	18.5 [14.8, 23.8]	18.6 [14.3, 23.6]	19.0 [13.8, 23.9]	20.4 [15.7, 25.0]	22.3 [17.3, 23.5]	21.6 [17.0, 23.0]	21.0 [16.1, 23.6]	21.6 [18.4, 25.6]
FM, %^b^	31.3 [24.0, 33.7]	30.3 [24.7, 33.8]	30.9 [24.9, 32.8]	30.7 [26.4, 35.3]	29.5 [26.0, 38.3]	29.1 [24.9, 37.8]	27.6 [25.1, 36.8]	29.9 [25.5, 37.9]
LL LBM, kg^a^	15.3 [14.5, 19.7]	15.5 [14.3, 19.1]	15.8 [14.5, 18.8]	16.3 [14.4, 18.5]	15.9 [14.4, 22.3]	15.6 [14.1, 21.0]	16.0 [14.8, 22.6]	17.7 [14.3, 23.0]
LL FM, kg^b^	6.6 [4.6, 8.7]	6.6 [4.9, 8.5]	7.4 [4.8, 9.2]	7.2 [5.1, 9.7]	6.7 [5.8, 8.6]	6.3 [5.8, 8.8]	6.3 [5.5, 8.6]	6.5 [5.5, 8.9]

*Note*: Data are presented as the median [interquartile range].

Abbreviations: BDC, baseline data collection; FM, fat mass; LBM, lean body mass; LL, lower limb; R, recovery.

^a^ Effect of time, *P *< 0.05,

^b^ Group × time interaction, *P *< 0.05.

#### 3.2.2 Fat mass

There were no main effects of time or group on total FM, lower‐limb FM and FM% (all *P*‐values ≥ 0.08). All three variables, however, showed a time‐by‐group interaction [total FM: *F*(3, 55) = 4.654, *P* = 0.005; leg FM: *F*(3, 55) = 5.559, *P* = 0.002; FM%: *F*(3, 55) = 5.707, *P* = 0.002, *n* = 22]. Contrasts showed that there was a statistically significant reduction in total FM in the exercise group after bed rest (R3) compared with baseline [BDC1; mean difference −0.65 kg, 95% CI (−0.99, −0.31), *P* = 0.001, *n* = 11]. Regarding leg FM, only the exercise group showed a reduction at the 4‐week follow‐up (R35) compared with baseline [mean difference −0.31 kg, 95% CI (−0.56, −0.053), *P* = 0.019].

### 3.3 Muscle volume and fat content

**FIGURE 4:**
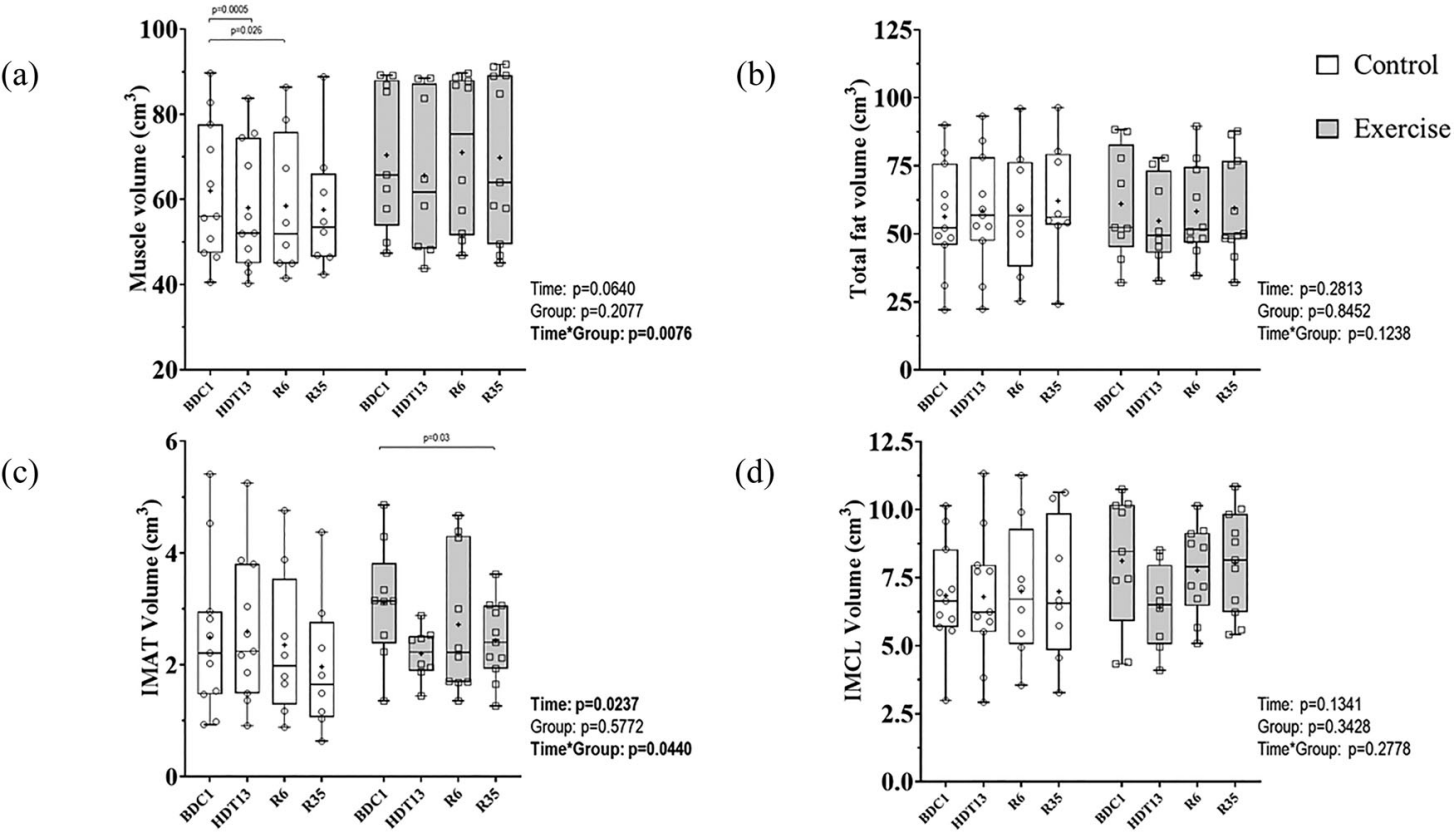
Thigh muscle volume and fat infiltration. Thigh muscle volume (a), total fat (b), intermuscular adipose tissue (IMAT; c) and intramyocellular lipids (IMCL; d) at baseline (BDC1), at the end of bed rest (HDT13), following 1 week of recovery (R6) and at the 4‐week follow‐up (R35) in the control group (*n* = 11; white boxes) and exercise groups (*n* = 9; grey boxes).

Muscle volume, total fat volume, intramyocellular lipid volume and intermuscular adipose tissue volume were measured with MRI (Figure 4). There was no main effect of time or group for muscle volume, but a group‐by‐time interaction was observed [*F*(3, 48) = 4.462, *P* = 0.0076, *n* = 22]. *Post hoc* analyses show that muscle volume was significantly reduced in the control group at the end of bed rest (HDT13) compared with baseline [BDC1; mean difference −3.99 cm^3^, 95% CI (−5.89, −2.09), *P* = 0.0005, *n* = 11], but not in the exercise group [mean difference −0.71 cm^3^, 95% CI (−2.23, 0.81), *P *= 0.40, *n* = 7]. Furthermore, muscle volume in the control group had not recovered back to baseline levels after 1 week of recovery (R6) compared with baseline [mean difference −2.15 cm^3^, 95% CI (−4.01, −0.30), *P *= 0.026, *n* = 8].

There was a main effect of time [*F*(1.655, 26.49) = 4.666, *P* = 0.024, *n* = 22] and a group‐by‐time interaction [*F*(3, 48) = 2.908, *P* = 0.044, *n *= 22] on intermuscular adipose tissue. Within‐group contrasts showed a reduction in intermuscular adipose tissue in the exercise group at the 4‐week follow‐up compared with baseline [mean difference −0.72 cm^3^, 95% CI (−1.37, −0.07), *P* = 0.032, *n* = 9]. There were no main effects of time, group or their interaction on total muscle fat volume or intramyocellular lipids (*P* ≥ 0.124 for all).

### 3.4 Muscle strength

Maximal handgrip strength and KES were measured four times during the study, and the data are presented in Table 3. There were no main effects of time or group, nor an interaction for handgrip strength (all *P*‐values ≥0.330). Analyses for KES showed a main effect of time [*F*(2.676, 48.17) = 11.13, *P* < 0.0001, *n* = 21], but no main effect of group or group‐by‐time interaction (all *P*‐values ≥ 0.407). *Post hoc* analyses showed a decrease in KES after bed rest (R2) compared with baseline [BDC5; mean difference −23.6 N m, 95% CI (−34.8, −12.4), *P* < 0.0001, *n* = 20], which represents a 13.4% decline in maximal voluntary contraction compared with baseline. This effect was no longer significant at the 4‐week follow‐up [R35; mean difference −9.4 N m, 95% CI (−22.6, 3.8), *P* = 0.202, *n* = 20].

**TABLE 3:** Muscle strength.

Variable	Control (HGS, *n* = 11; KES, *n* = 10)				Exercise (*n* = 11)			
	BDC	R1‐2	R35	R120	BDC	R1‐2	R35	R120
HGS, kg	68 [61, 93]	67 [60, 101]	75 [62, 99]	78 [65, 99]	72 [54, 114]	66 [58, 121]	68 [60, 124]	74 [60, 116]
KES, N m^a^	140 [121, 228]	136 [100, 178]	140 [101, 183]	154 [116, 168]	199 [108, 220]	181 [114, 202]	180 [118, 214]	187 [125, 238]

*Note*: Data are presented as the median [interquartile range].

Abbreviations: BDC, baseline data collection; HGS, handgrip strength; KES, knee‐extensor strength, R, recovery.

^a^ Effect of time, *P *< 0.0001.

**FIGURE 5:**
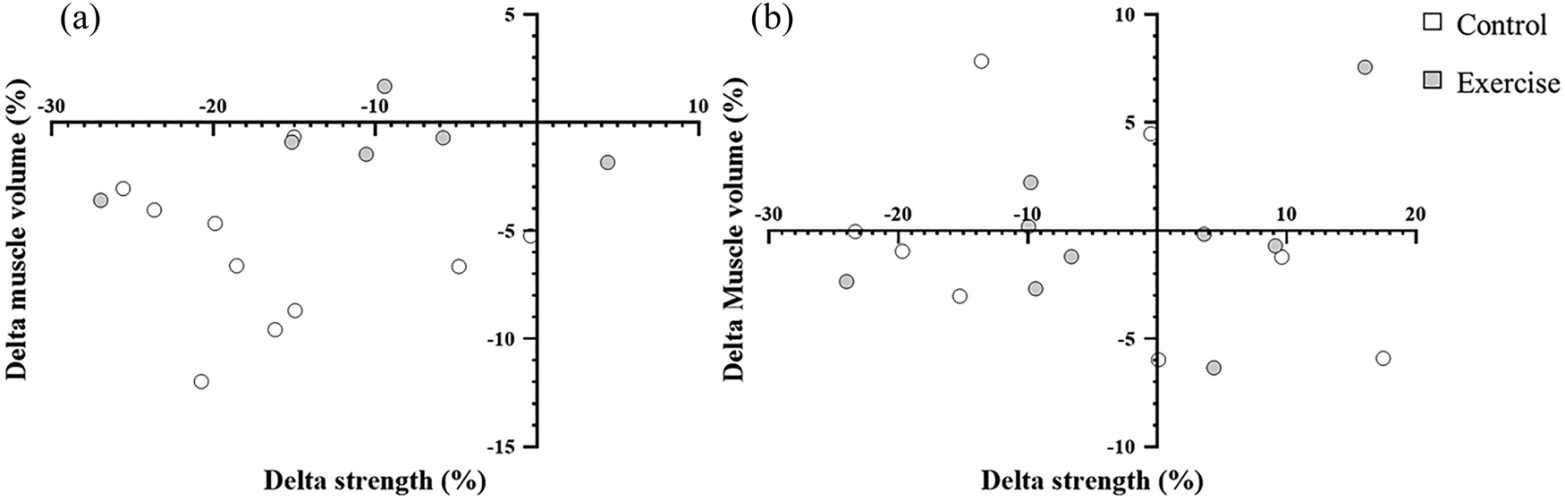
Relationship between changes in muscle volume and strength. Relationship between the relative changes compared with baseline levels in muscle volume and muscle strength after 2 weeks of bed rest (a; *n* = 16) and at the 4‐week follow‐up (b; *n* = 17). The Spearman analyses were conducted in the pooled sample, but participants in the control group are represented in white and participants in the exercise group are represented in grey.

Interestingly, the changes in KES were not correlated with the changes in thigh muscle volume after bed rest [ρ_s _= 0.23, 95% CI (−0.32, 0.66), *P* = 0.398, *n* = 16] nor after 4 weeks [ρ_s _= –0.15, 95% CI (−0.60, 0.37), *P* = 0.559, *n* = 17]. The data are presented in Figure 5.

### 3.5 Muscle quality

**FIGURE 6:**
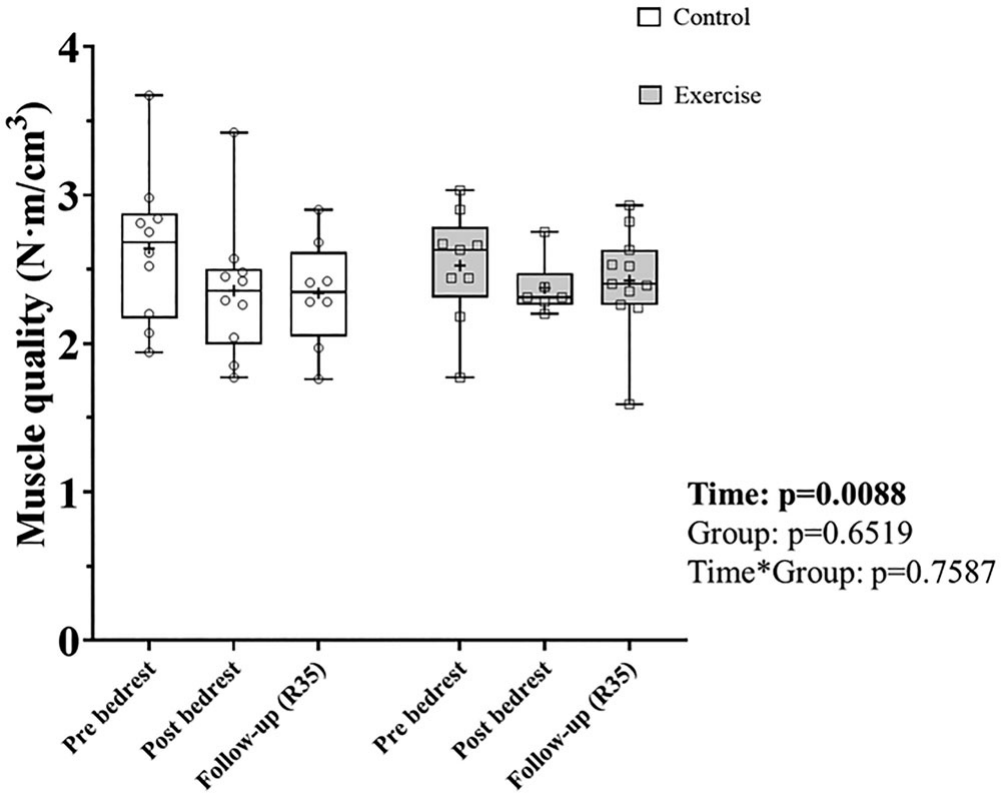
Muscle quality, defined as the ratio of knee‐extensor maximal isometric force (in newton metres) to thigh muscle volume (in centimetres cubed), in the control group (*n* = 10; white boxes) and exercise group (*n* = 9; grey boxes).

Muscle quality was determined as the ratio of KES divided by thigh muscle volume, as illustrated in Figure 6. There was a main effect of time [*F*(1.634, 23.69) = 6.391; *P* = 0.009, *n* = 21], without a main effect of group or a time‐by‐group interaction (all *P*‐values ≥ 0.652). Concordant with previous KES results, a significant reduction was observed after bed rest [mean change −0.28 N m/cm^3^, 95% CI (−0.43, −0.13), *P* < 0.001, *n* = 16] and was no longer significantly different from baseline at the 4‐week follow‐up [R35; mean change −0.11 N m/cm^3^, 95% CI (−0.32, 0.10), *P* = 0.353, *n* = 17].

### 3.6 Muscle proteomics

A total of 3344 proteins were identified across the different time points. Of those, 2999 passed the inclusion filter and were included in the final differential expression analysis, and 3061 were included in the supplementary analyses. The number of proteins that were up‐ and downregulated at each time point in both groups are presented in Figure S1. Volcano plots for up‐ and downregulated proteins and associated biological processes are presented in Figures S2–S9 for each group.

**FIGURE 7:**
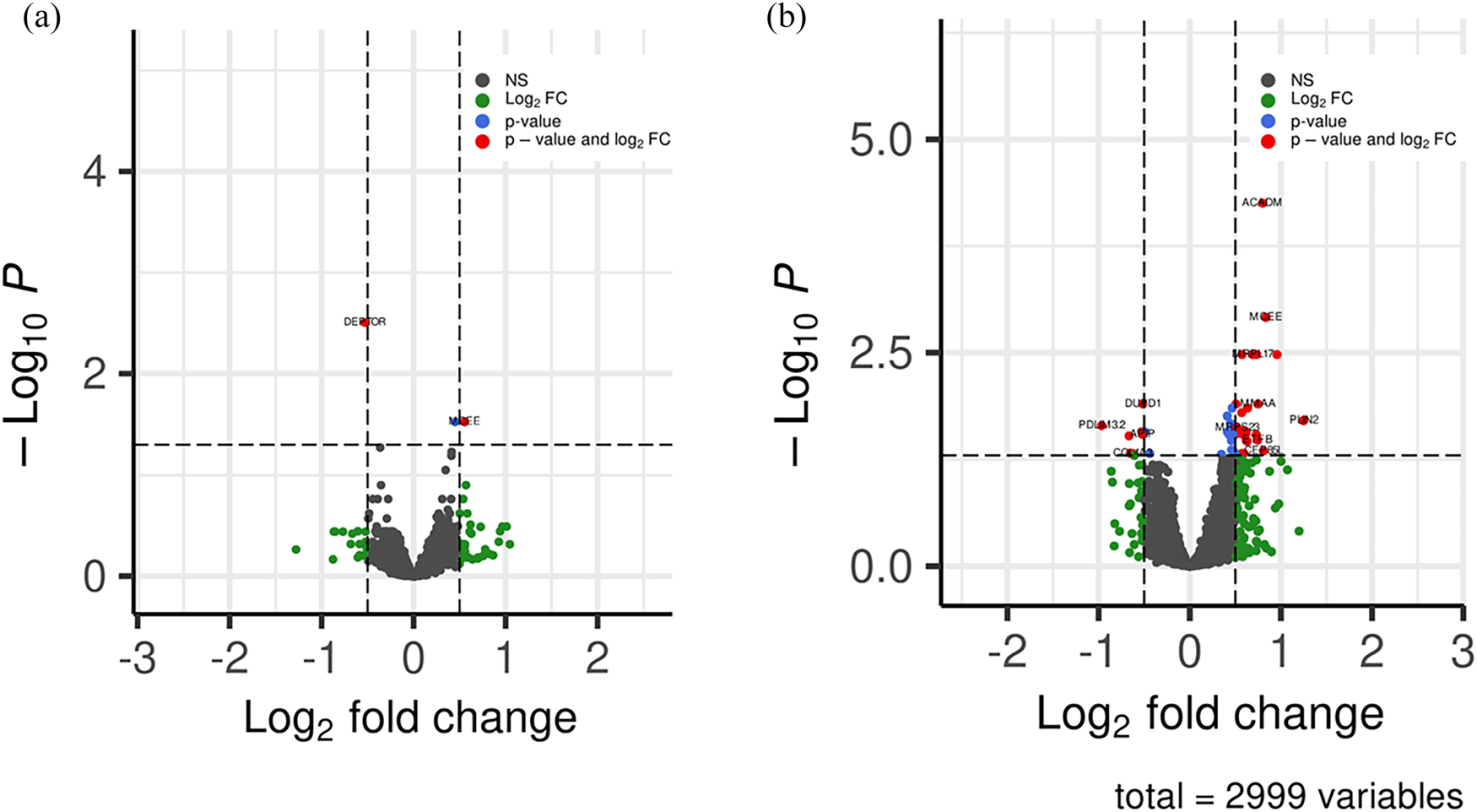
Volcano plots of differentially expressed proteins between groups during (a; HDT8) and after (b; R7) bed rest. In the volcano plots, proteins shown in red are significantly differentially expressed, meeting both the *P*‐value and fold‐change criteria. Proteins in blue meet only the *P*‐value threshold, whereas those in green meet only the fold‐change threshold.

**FIGURE 8:**
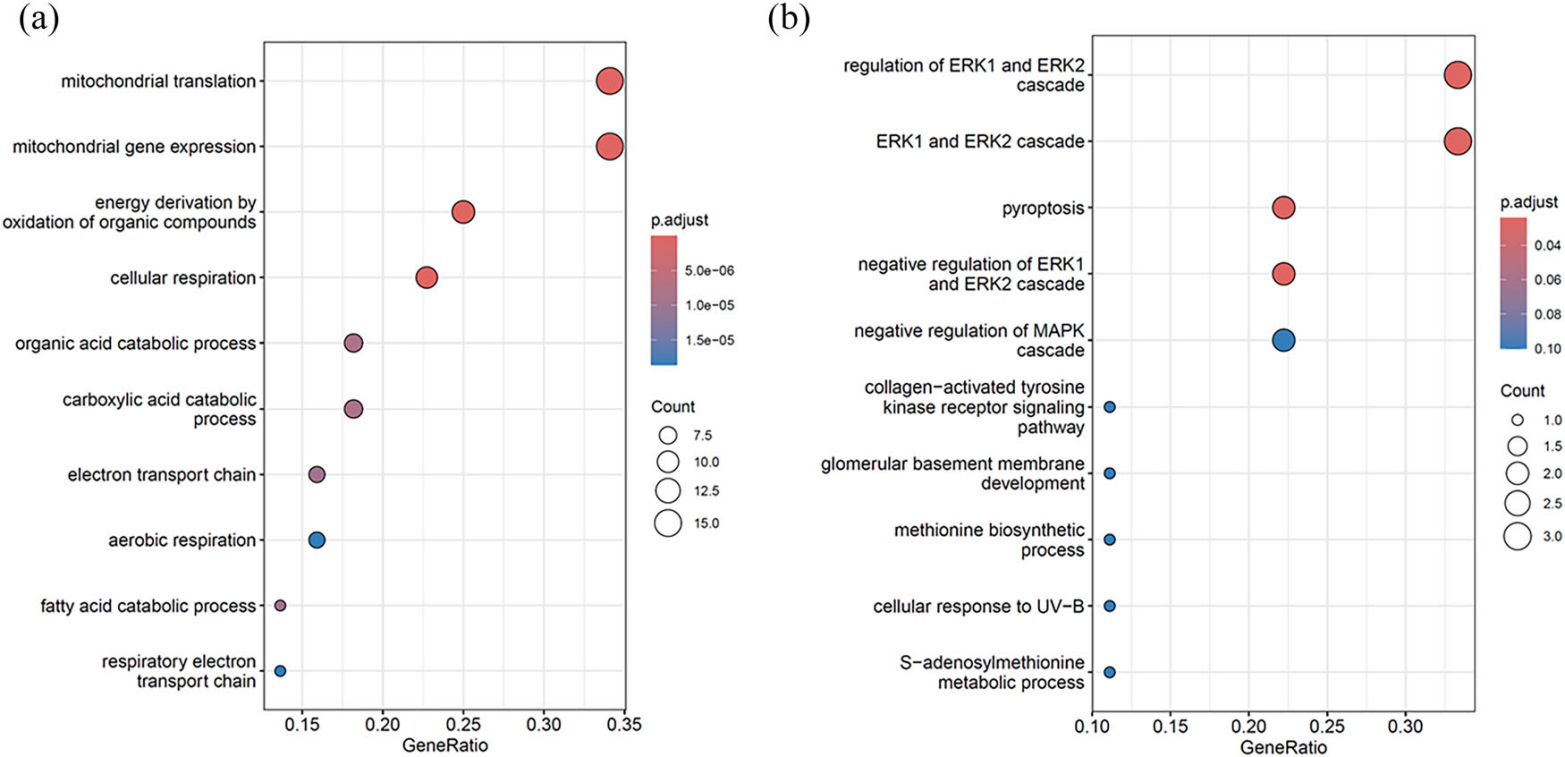
Biological processes associated with the differentially expressed proteins between groups after bed rest (R7). (a) Processes that were upregulated in the exercise group compared with the control group. (b) Processes that were downregulated in the exercise compared with the control group.

Between‐group contrasts revealed no significant differences at baseline (HDT1), during early bed rest (HDT3) or at the 4‐week follow‐up (R35). At HDT8, the only time point available during bed rest in the primary analyses, two proteins were differentially expressed between groups (Figure 7a). DEPTOR (*DEP domain‐containing mTOR‐interacting protein*; also known as DEPDC6), an endogenous inhibitor of mTORC1, was significantly downregulated in the exercise group compared with controls, whereas MCEE (*Methylmalonyl CoA epimerase*), a protein involved in fatty acid catabolism, was upregulated. After 1 week of recovery (R7), 37 proteins were differentially expressed between groups, and 54 proteins met the adjusted *P*‐value threshold (Figure 7b). Gene ontology analysis further indicated that proteins involved in mitochondrial translation and mitochondrial gene expression were upregulated in the exercise group compared with the control group (Figure 8). Supplementary analyses for late bed rest (HDT14) showed similar observations, with proteins associated with mitochondrial translation and gene expression being upregulated in the exercise group (Figure S10). Interestingly, the DEPTOR protein identified at HDT8 was still differentially expressed at HDT14.

Within‐group GO enrichment analyses revealed that in the control group, the predominant biological processes associated with the proteins downregulated during bed rest (HDT3 and HDT8) were muscle contraction, endosomal transport, regulation of protein stability, basement membrane organization and integrin‐mediated signalling. Conversely, processes associated with upregulated proteins included transmembrane transporter activity, muscle system processes (a broad gene ontology term encompassing muscle contraction/relaxation, development and adaptation; *GO:0003012*) and cytoplasmic translation. In the exercise group, bed rest was characterized by downregulation of muscle system processes and muscle cell differentiation, whereas nuclear transport, RNA splicing and cellular respiration were upregulated.

During the recovery period (R7 and R35), the control group showed downregulation of mitochondrial translation and gene expression, muscle contraction, muscle system processes and cellular respiration. In contrast, cytoplasmic translation, RNA splicing, ribonucleoprotein and ribosome biogenesis and protein folding were upregulated. In the exercise group, recovery was marked by downregulation of muscle system processes, muscle contraction, cytoplasmic translation and metabolic processes, alongside upregulation of cytoplasmic translation, cellular respiration, RNA splicing, ribonucleoprotein and ribosome biogenesis and protein folding pathways.

### 3.7 Plasma inflammatory markers

Selected inflammatory markers were measured in plasma after an overnight fast, and the results are presented in Table 4. There were no main effects of time, group or time‐by‐group interactions with any of the three inflammatory markers (all *P*‐values ≥0.085).

**TABLE 4:** Plasma cytokines.

Variable	Control (IL‐6, *n* = 6; IL‐8, *n* = 9; TNF‐α, *n* = 9)					Exercise (IL‐6, *n* = 10; IL‐8, *n* = 11; TNF‐α, *n* = 11)				
	BDC1	HDT14	R7	R35	R120	BDC1	HDT14	R7	R35	R120
IL‐6, pg/mL	0.9 [0.2, 14.5]	1.7 [0.6, 15.1]	7.2 [0.3, 14.5]	1.0 [0.4, 14.1]	1.5 [0.6, 13.3]	2.6 [0.7, 12.9]	2.7 [1.2, 16.4]	3.0 [0.8, 16.3]	1.9 [0.9, 6.7]	3.3 [1.1, 13.3]
IL‐8, pg/mL	5.4 [3.3, 13.2]	5.6 [3.1, 18.0]	5.4 [2.5, 15.6]	4.8 [2.7, 16.5]	4.8 [2.9, 12.0]	5.6 [3.8, 15.1]	5.9 [3.1, 16.0]	6.1 [3.3, 17.3]	5.7 [3.0, 9.9]	5.5 [3.3, 14.4]
TNF‐α, pg/mL	7.3 [5.2, 8.5]	7.1 [5.0, 8.5]	6.6 [5.1, 8.7]	6.8 [5.2, 8.5]	7.0 [5.2, 9.2]	6.7 [5.4, 8.3]	7.1 [5.9, 9.8]	7.3 [5.6, 8.1]	7.2 [5.5, 9.4]	6.6 [5.8, 7.9]

*Note*: Data are presented as the median [interquartile range].

Abbreviations: BDC, baseline data collection; HDT, head‐down tilt bed rest; IL, interleukin; R, recovery; TNF, tumour necrosis factor.

## 4 DISCUSSION

We examined the effects of 14 days of head‐down bed rest and a mixed‐exercise countermeasure on alterations in muscle proteomics, body composition, muscle volume, strength, quality and inflammatory markers in older adults. Our findings revealed an overall decline in total and lower‐limb LBM, KES and muscle quality following the 14 day head‐down bed rest period, which had returned to baseline levels after 4 weeks of recovery. We also observed an impact of the mixed‐exercise intervention on muscle proteomics and thigh muscle volume. This study represents one of the first investigations to explore the effects of an exercise countermeasure in healthy ageing adults subjected to a period of head‐down bed rest.

### 4.1 Muscle proteomics

Our primary proteomic analyses revealed a selective between‐group response during bed rest, with only two proteins, MCEE and DEPTOR, being differentially expressed. In contrast, gene ontology analyses during early recovery indicated a broader proteomic response, with enrichment of processes related to cellular respiration, mitochondrial translation and mitochondrial gene expression in the exercise group compared with the control group. Supplemental proteomic analyses during late bed rest also revealed an increased expression of proteins associated with mitochondrial translation, in addition to mitochondrial gene expression in the exercise group compared with the control group.

Given its established role as an endogenous inhibitor of mTORC1, the main regulator of muscle protein synthesis, and its previously suggested involvement in the regulation of LBM during disuse, DEPTOR was selected for focused discussion (Kazi et al., 2011; Mallinson & Murton, 2013; Roberson et al., 2020; Rosa‐Caldwell et al., 2021). In rodent models, DEPTOR protein and mRNA expression were increased following prolonged hindlimb unloading (48–168 h), but not during early immobilization (≤24 h) (Roberson et al., 2020; Rosa‐Caldwell et al., 2021). Furthermore, partial in vivo knockdown of DEPTOR has been shown to prevent muscle atrophy following 3 days of hindlimb immobilization in mice (Kazi et al., 2011). Consistent with these observations, we observed that thigh muscle atrophy was prevented in the exercise group alongside reduced DEPTOR protein expression during mid to late bed rest (HDT8 and HDT14), but not during early bed rest (HDT3). To the best of our knowledge, only one human study has examined the effect of immobilization on DEPTOR signalling, reporting no significant change after 10 days of unilateral leg immobilization in young women (Boileau, 2025). This aligns with our findings in the control group, in which DEPTOR expression remained unchanged throughout bed rest. These results suggest that DEPTOR regulation during disuse might differ between humans and animal models and highlight the need for further studies to elucidate the effects of bed rest and exercise countermeasures on DEPTOR protein expression and downstream signalling activity.

Bed rest and immobilization are known to affect mitochondrial density and function negatively (Deane et al., 2021; Eggelbusch et al., 2024) which, in turn, might contribute to disuse‐induced muscle atrophy (Kang & Ji, 2016; Romanello & Sandri, 2021). For instance, Eggelbusch et al. (2024) reported that 55 days of bed rest resulted in a 24% reduction in mitochondrial respiration and a 30% decrease in mitochondrial density, paralleled by a significant decline in fat free mass. These findings are supported by mechanistic evidence showing that overexpression of PGC‐1α, a master regulator of mitochondrial biogenesis, protected against disuse‐induced muscle atrophy in rodent models (Kang & Ji, 2016). In line with this literature, our between‐group proteomic analyses revealed increased mitochondrial translation activity and gene expression in the exercise group compared with control group during late bed rest (HDT14; Supporting Information) and early recovery (R6). Importantly, these observations coincided with the preservation of thigh muscle volume in the exercise group, whereas the control group remained below baseline values. Together, these findings suggest that exercise‐induced mitochondrial remodelling might help to attenuate disuse‐related muscle loss at the local level in bedridden older adults.

### 4.2 Changes in body composition and muscle volume

During the 14‐day bed rest period, there was a statistically significant decrease in total and lower‐limb LBM as assessed by DXA, but no significant impact of the mixed‐exercise intervention. It is important to point out, however, that average relative changes of LBM and leg LBM fell within the reported coefficient of variation of 2% for LBM measured by DXA (Hangartner et al., 2013). Hence, although the statistical tests indicate a significant effect of time, these results should be interpreted with caution. In contrast, an interaction effect was observed for thigh muscle volume measured by MRI, with only the control group showing a reduction in volume. This observation suggests a potential mitigating effect of exercise.

Previous research has demonstrated a decline in LBM among older adults after bed rest (Di Girolamo et al., 2021; Kortebein et al., 2007; Pisot et al., 2016). In a meta‐analysis, Di Girolamo et al. (2021) reported average reductions of 5.5% and 8.5% in total and lower‐limb LBM, respectively, following a 14 day bed rest period. These findings exceed the 1.5% and 2.0% decreases observed in our study for total and lower‐limb LBM. Even when considering only the control group, the reductions in total and lower‐limb LBM were found to be 1.8% and 2.1%, respectively. The disparities between our observations and those from previous literature might be attributed to the consumption by participants of a high‐protein diet (>1.2 g/kg/day). Indeed, recent studies have provided evidence indicating that a high‐protein diet might protect LBM during the first 2 weeks of bed rest (Gao & Chilibeck, 2020; Vinci et al., 2022). Furthermore, it was posited that increased protein intake can limit disuse‐induced muscle loss, especially when baseline dietary protein intake was low to begin with (Stein & Blanc, 2011). As a result, the dietary prescription implemented in our study might have hindered our capacity to observe the effects of an exercise intervention.

The inconsistencies observed between the two imaging modalities could be attributed to two primary factors. Firstly, it cannot be excluded that DXA lacks the sensitivity to measure subtle changes over time properly. A study by Maden‐Wilkinson et al. (2013) showed that DXA underestimated the loss of muscle mass in older adults compared with MRI measurements. Secondly, the inconsistencies between the imaging modalities could reflect the divergence in muscles assessed. Indeed, the MRI images are limited to the thigh muscles, whereas DXA measured the whole lower‐limb LBM. In their study in younger women, Trappe et al. (2007) demonstrated that an exercise intervention successfully prevented the decline in muscle volume specifically in the quadriceps femoris muscle, whereas the triceps surae muscle was not protected to the same extent (Trappe et al., 2007). Consequently, it is plausible that solely evaluating muscle volume in the thigh muscles might misrepresent the broader impact of the exercise intervention.

To the best of our knowledge, only one other study has examined the impact of an exercise countermeasure during bed rest in older adults. The countermeasure, consisting of 2000 steps/day, was found to be insufficient to mitigate the loss of total or lower‐limb LBM (Arentson‐Lantz et al., 2019). Our findings align with these results and indicate that physical activity and exercise alone might not be sufficient in countering the decline of LBM caused by prolonged bed rest in ageing adults. Interestingly, alternative approaches, such as neuromuscular electrical stimulation, have been used to lessen the negative effects of muscle disuse in older adults. In a study conducted by Reidy et al. (2017), application of 40 min of neuromuscular electrical stimulation three times per day was effective in preventing muscle atrophy, but not loss of strength during a 5 day bed rest period. Further studies looking at the combined effect of exercise and alternative approaches, such as neuromuscular electrical stimulation, could be interesting.

### 4.3 Changes in muscle strength and muscle quality

The period of bed rest resulted in a decline in muscle quality and isometric maximal KES, although maximal handgrip strength remained unaffected. Although no interaction effect was observed, within‐group analyses demonstrated that the control group displayed a decrease in muscle quality and KES following bed rest, whereas exercising appeared to attenuate this impact. Both muscle quality and KES recovered to baseline values after a 4 week period. The lack of correlation between changes in KES and muscle volume agrees with previous studies and suggests that other mechanisms could be at play, such as reduced fibre specific tension or reduced voluntary activation (Campbell et al., 2019; Marusic et al., 2021).

Referring back to the study by Arentson‐Lantz et al. (2019), a 2000 steps/day intervention was insufficient to counteract the loss of strength induced by bed rest. The relative decrease in strength observed by these authors was ∼12.0%, which is similar to our pooled sample (−11.6%). Again, within‐group analyses revealed a relative decrease in KES of −16.5% and −6.7% for the control and exercise groups, respectively, suggesting that the countermeasure might have played a role in mitigating the impact of bed rest on muscle strength. Furthermore, and consistent with our findings, Rejc et al. (2018) demonstrated that older adults fully regained their maximal voluntary strength within 2 weeks after a 14 day bed rest period. Similar results were also obtained by Pisot et al. (2016), who showed that 2 weeks of rehabilitation were sufficient to recover KES in older adults subjected to 14 days of bed rest. Finally, the lack of significant change in handgrip strength was anticipated in our study. In their comprehensive review on the subject, Kehler et al. (2019) highlighted that head‐down bed rest of ≤8 weeks primarily impacts the lower limbs, with minimal to no alterations observed in the upper limbs. These outcomes could potentially be attributed to the involvement of participants in various activities requiring their hands throughout the day.

### 4.4 Changes in inflammatory markers

Despite changes in muscle mass and muscle volume, no changes were observed in circulating inflammatory markers in either group. The absence of changes in circulating inflammatory markers is consistent with the proteomic profile, which did not indicate strong enrichment of inflammatory pathways, suggesting that muscle adaptations that occurred were largely independent of systemic inflammation. Nevertheless, it should be acknowledged that only three circulating cytokines were assessed, which might not fully capture the complexity of the inflammatory response, particularly at the level of skeletal muscle.

IL‐6, IL‐8 and TNF‐α are cytokines commonly implicated in inflammatory and catabolic processes associated with muscle atrophy and muscle wasting in the context of ageing and disease (Cruz et al., 2024; He et al., 2024; Sartori et al., 2021). However, it remains unclear whether immobilization and bed rest elicit a pro‐inflammatory response in older adults, because previous studies have reported inconsistent findings (Bosutti et al., 2008; Drummond et al., 2013; Jurdana et al., 2015). For instance, Jurdana et al. (2015) reported increased serum IL‐6 and TNF‐α levels after a 14 day bed rest period in older adults. In contrast, and in agreement with our results, Drummond et al. (2013) reported no significant changes in circulating IL‐6, IL‐8 or TNF‐α after a 7 day bed rest period, with most other measured serum inflammatory markers also remaining unchanged. Interestingly, despite the absence of systemic inflammation, Drummond et al. (2013) observed elevated muscle IL‐6 mRNA expression, suggesting the presence of localized inflammatory signalling within skeletal muscle. However, this IL‐6 mRNA expression was not associated with changes in leg lean mass.

The discrepancy between our findings and those reported by Jurdana et al. (2015) might be explained, in part, by differences in baseline inflammatory status. In our cohort, baseline circulating IL‐6 concentrations were generally low (median: control, 0.9 pg/mL; exercise, 2.6 pg/mL), whereas participants in the study by Jurdana et al. (2015) exhibited substantially higher baseline levels (mean ∼17.5 pg/mL). Circulating IL‐6 concentrations exceeding 2.5 pg/mL have been proposed as an indication of chronic low‐grade inflammation (Ferrucci et al., 1999) and might predispose individuals to a more pronounced systemic inflammatory response to bed rest.

### 4.5 Strengths and limitations of the study

One notable strength of this study lies in the unique population and exercise countermeasure investigated. Considering the potential adverse effects of head‐down bed rest on participants, previous studies have predominantly focused on younger individuals with greater physiological resilience and capacity for recovery. However, our study stands out as one of the first to examine the effects of head‐down bed rest specifically in individuals aged ≥55 years, who are more susceptible to experience bed riddance and might be subjected to some ageing‐associated muscle adaptations that would influence underlying mechanisms.

We recognize that this study has some limitations. One of the primary limitations is the statistical power. Some participants either dropped out or were unable to complete certain tests, potentially diminishing our ability to observe differences between the groups. To mitigate this limitation, we tried to report as much information as possible for the reader to be able to analyse the results critically. It is worth noting that a final sample size of 22 participants across two groups is typical in the context of bed rest studies. The timing of measurements is a second limit to the study. Considering the extensive nature of the project, it was not feasible to conduct all tests on the initial day of recovery. Consequently, strength measurements were taken on the second day following bed rest, and LBM was assessed using DXA on the third day, potentially allowing for some degree of recovery. There were no statistical between‐group differences for energy intake during the bed rest period, nor within‐group differences for energy intake between ambulatory and bed‐rest periods (Guan, 2022). This could either suggest that the control group was in caloric excess or that the exercise group was in caloric deficit during bed rest, and both scenarios are likely to have influenced body composition results. Finally, we acknowledge that high‐intensity exercise might not be feasible for all older adults, especially in the context of hospitalization, hence the intervention used herein might not be applicable in its present form.

## 5 CONCLUSION

In summary, our study demonstrates that a daily 1 h mixed‐exercise programme effectively preserved the volume of thigh muscles during a 14 day period of head‐down bed rest in healthy ageing adults. However, this exercise intervention was insufficient to counteract the negative impact of bed rest on total and lower‐limb lean body mass, KES and muscle quality. A 4 week recovery period was sufficient to restore all impairments caused by bed rest fully, except for thigh muscle volume in the control group. Further investigation is warranted to assess the translation of our intervention to a hospitalized population, considering that individuals with underlying health conditions might experience more pronounced impairments in comparison to those observed in our study.

## AUTHOR CONTRIBUTIONS

Conception of the study: Isabelle J. Dionne, Eléonor Riesco, Karen Lambert, Jamie S. McPhee, Jose A. Morais and Ahmed Ghachem Data acquisition: Jean‐Christophe Lagacé, Philippe St‐Martin and Guy Hajj‐Boutros Data analysis: Jean‐Christophe Lagacé, Mariano Avino and Guy Hajj‐Boutros Manuscript draft: Jean‐Christophe Lagacé Critical manuscript revision: all authors. All authors approved the final version of the manuscript and agree to be accountable for all aspects of the work in ensuring that questions related to the accuracy or integrity of any part of the work are appropriately investigated and resolved. All persons designated as authors qualify for authorship, and all those who qualify for authorship are listed.

## CONFLICT OF INTEREST

None declared.

## Supporting information




**FIGURE S1** Number of differentially expressed proteins compared with baseline. *Note*: The numbers of proteins up‐ (black bars) and downregulated (grey bars) are presented for the control group (left side of the figure) and the exercise group (right side of the figure) compared with their respective baseline (HDT1). In total, 2999 proteins were identified and included in the analyses for each time point. Differentially expressed proteins were defined as those with a log_2_ fold‐change ≥ 0.5 and a false discovery rate‐adjusted *P*‐value ≤ 0.05.

**FIGURE S2** Volcano plot and gene ontology biological processes changes between HDT3 and HDT1 in the control group. *Note*: Frame A represents the volcano plot of proteins that are differentially expressed between HDT3 and HDT1 in the control group. In the volcano plots, proteins shown in red are significantly differentially expressed, meeting both the *P*‐value and fold‐change criteria. Proteins in blue meet only the *P*‐value threshold, whereas those in green meet only the fold‐change threshold. Frame B represents the biological processes associated with upregulated proteins, and Frame C represents the biological processes associated with downregulated proteins.

**FIGURE S3** Volcano plot and gene ontology biological processes changes between HDT8 and HDT1 in the control group. *Note*: Frame A represents the volcano plot of proteins that are differentially expressed between HDT8 and HDT1 in the control group. In the volcano plots, proteins shown in red are significantly differentially expressed, meeting both the *P*‐value and fold‐change criteria. Proteins in blue meet only the *P*‐value threshold, whereas those in green meet only the fold‐change threshold. Frame B represents the biological processes associated with upregulated proteins, and Frame C represents the biological processes associated with downregulated proteins.

**FIGURE S4** Volcano plot and gene ontology biological processes changes between R7 and HDT1 in the control group. *Note*: Frame A represents the volcano plot of proteins that are differentially expressed between R7 and HDT1 in the control group. In the volcano plots, proteins shown in red are significantly differentially expressed, meeting both the *P*‐value and fold‐change criteria. Proteins in blue meet only the *P*‐value threshold, whereas those in green meet only the fold‐change threshold. Frame B represents the biological processes associated with upregulated proteins, and Frame C represents the biological processes associated with downregulated proteins.

**FIGURE S5** Volcano plot and gene ontology biological processes changes between R35 and HDT1 in the control group. *Note*: Frame A represents the volcano plot of proteins that are differentially expressed between R35 and HDT1 in the control group. In the volcano plots, proteins shown in red are significantly differentially expressed, meeting both the *P*‐value and fold‐change criteria. Proteins in blue meet only the *P*‐value threshold, whereas those in green meet only the fold‐change threshold. Frame B represents the biological processes associated with upregulated proteins, and Frame C represents the biological processes associated with downregulated proteins.

**FIGURE S6** Volcano plot and gene ontology biological processes changes between HDT3 and HDT1 in the exercise group. *Note*: Frame A represents the volcano plot of proteins that are differentially expressed between HDT3 and HDT1 in the exercise group. In the volcano plots, proteins shown in red are significantly differentially expressed, meeting both the *P*‐value and fold‐change criteria. Proteins in blue meet only the *P*‐value threshold, whereas those in green meet only the fold‐change threshold. Frame B represents the biological processes associated with upregulated proteins, and Frame C represents the biological processes associated with downregulated proteins.

**FIGURE S7** Volcano plot and gene ontology biological processes changes between HDT8 and HDT1 in the exercise group. *Note*: Frame A represents the volcano plot of proteins that are differentially expressed between HDT8 and HDT1 in the exercise group. In the volcano plots, proteins shown in red are significantly differentially expressed, meeting both the *P*‐value and fold‐change criteria. Proteins in blue meet only the *P*‐value threshold, whereas those in green meet only the fold‐change threshold. Frame B represents the biological processes associated with upregulated proteins, and Frame C represents the biological processes associated with downregulated proteins.

**FIGURE S8** Volcano plot and gene ontology biological processes changes between R7 and HDT1 in the exercise group. *Note*: Frame A represents the volcano plot of proteins that are differentially expressed between R7 and HDT1 in the exercise group. In the volcano plots, proteins shown in red are significantly differentially expressed, meeting both the *P*‐value and fold‐change criteria. Proteins in blue meet only the *P*‐value threshold, whereas those in green meet only the fold‐change threshold. Frame B represents the biological processes associated with upregulated proteins, and Frame C represents the biological processes associated with downregulated proteins.

**FIGURE S9** Volcano plot and gene ontology biological processes changes between R35 and HDT1 in the exercise group. *Note*: Frame A represents the volcano plot of proteins that are differentially expressed between R35 and HDT1 in the exercise group. In the volcano plots, proteins shown in red are significantly differentially expressed, meeting both the *P*‐value and fold‐change criteria. Proteins in blue meet only the *P*‐value threshold, whereas those in green meet only the fold‐change threshold. Frame B represents the biological processes associated with upregulated proteins, and Frame C represents the biological processes associated with downregulated proteins.

**FIGURE S10** Volcano plot and gene ontology biological processes differences between groups at HDT14. *Note*: Frame A represents the volcano plot of proteins that are differentially expressed between groups at HDT14. In the volcano plots, proteins shown in red are significantly differentially expressed, meeting both the *P*‐value and fold‐change criteria. Proteins in blue meet only the *P*‐value threshold, whereas those in green meet only the fold‐change threshold. Frame B represents the biological processes associated with upregulated proteins in the exercise compared with control group, and Frame C represents the biological processes associated with downregulated proteins in exercise compared with the control group.

## Data Availability

Data are available upon reasonable request.

## APPENDIX 1

### Distribution of training sessions during the bed rest period

**Table:**

Week 1	Day 1	Day 2	Day 3	Day 4	Day 5	Day 6	Day 7
Session 1	Resistance Upper body	Aerobic Continuous (30)	Aerobic Progressive	Aerobic Continuous (30)	HIIT	Aerobic Continuous (30)	HIIT
Session 2	Aerobic Progressive	Resistance Lower body	Aerobic Continuous (15)	Resistance Upper body	Aerobic Progressive	Resistance Upper body	Aerobic Continuous (15)
Session 3	HIIT	Aerobic Progressive	HIIT	Aerobic Progressive	Resistance Lower body	Aerobic Progressive	Aerobic Progressive
Week 2	Day 8	Day 9	Day 10	Day 11	Day 12	Day 13	Day 14
Session 1	Aerobic Continuous (15)	HIIT	Aerobic Continuous (30)	HIIT	Aerobic Continuous (30)	HIIT	Aerobic Continuous (15)
Session 2	Resistance Upper body	Aerobic Continuous (15)	Resistance Lower body	Aerobic Continuous (15)	Resistance Lower body	Aerobic Progressive	Aerobic Continuous (30)
Session 3	Aerobic Progressive	Aerobic Progressive	Aerobic Progressive	Aerobic Progressive	Aerobic Progressive	Resistance Upper body	Aerobic Progressive

#### Details of the aerobic sessions

Continuous (30 min):

- 5 min warm‐up (40%–50% of heart rate reserve)
- 20 min continuous exercise (60%–70% of heart rate reserve)
- 5 min cool‐down (40%–50% of heart rate reserve).

Continuous (15 min):

- 3 min warm‐up (40%–50% of heart rate reserve)
- 9 min continuous exercise (60%–70% of heart rate reserve)
- 3 min cool‐down (40%–50% of heart rate reserve).

Progressive:

- Five 3 min stages performed at 30%, 40%, 50%, 60% and 40% of heart rate reserve.

High‐intensity interval training (HIIT):

- 5 min warm‐up (40%–50% of heart rate reserve)
- 11 alternations of 30 s at high intensity (80%–90% of heart rate reserve) and 90 s at low intensity
- 5 min cool‐down (40%–50% of heart rate reserve).

#### Details of the resistance sessions

Upper‐body resistance::

- Three sets (including one warm‐up set) of 10–12 repetitions of the following exercises: shoulder external rotation, chest fly and lateral pull‐down using a pulley station.

Lower‐body resistance:

- Three sets (including one warm‐up set) of 10–12 repetitions of the following exercises: hip raises, leg press, leg curls and ankle flexion/extension movements.
