# Supplementary figures and images for: Impact of 14 days of head‐down bed rest and an exercise countermeasure on skeletal muscle atrophy, proteome and circulatory cytokines in older adults
Source: Exp Physiol. 2026 Mar 27:10.1113/EP093524. Online ahead of print. doi: 10.1113/EP093524 (PMC13394170; doi:10.1113/EP093524)

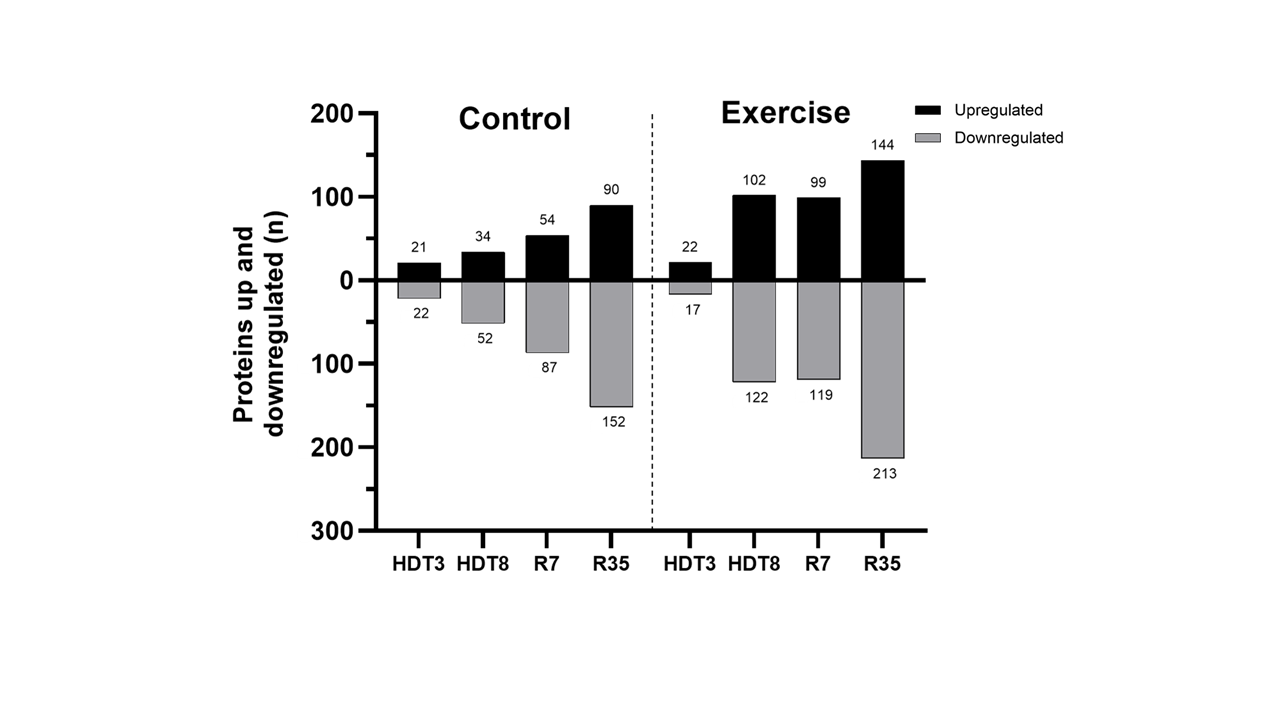

Supplement: Supplementary file 1 — FIGURE S1 Number of differentially expressed proteins compared with baseline. Note: The numbers of proteins up‐ (black bars) and downregulated (grey bars) are presented for the control group (left side of the figure) and the exercise group (right side of the figure) compared with their respective baseline (HDT1). In total, 2999 proteins were identified and included in the analyses for each time point. Differentially expressed proteins were defined as those with a log2 fold‐change ≥ 0.5 and a false discovery rate‐adjusted P‐value ≤ 0.05. [file EPH-9999-0-s002.tif]

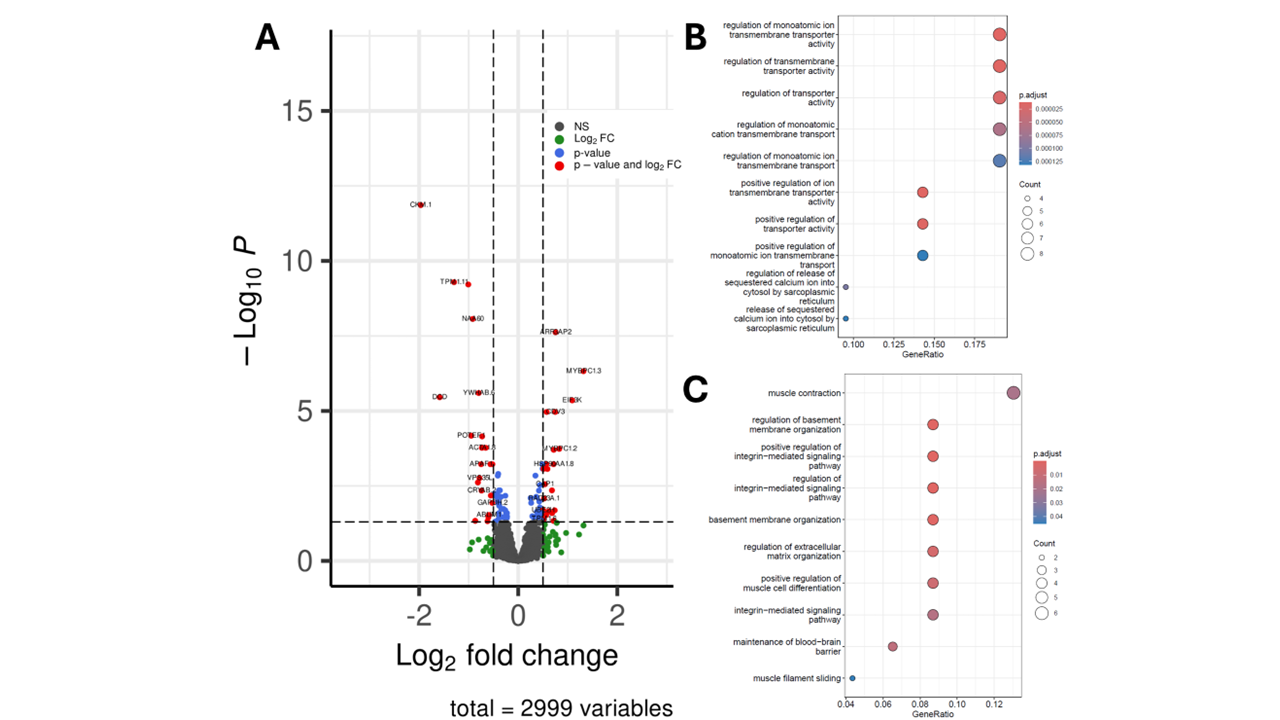

Supplement: Supplementary file 2 — FIGURE S2 Volcano plot and gene ontology biological processes changes between HDT3 and HDT1 in the control group. Note: Frame A represents the volcano plot of proteins that are differentially expressed between HDT3 and HDT1 in the control group. In the volcano plots, proteins shown in red are significantly differentially expressed, meeting both the P‐value and fold‐change criteria. Proteins in blue meet only the P‐value threshold, whereas those in green meet only the fold‐change threshold. Frame B represents the biological processes associated with upregulated proteins, and Frame C represents the biological processes associated with downregulated proteins. [file EPH-9999-0-s003.tif]

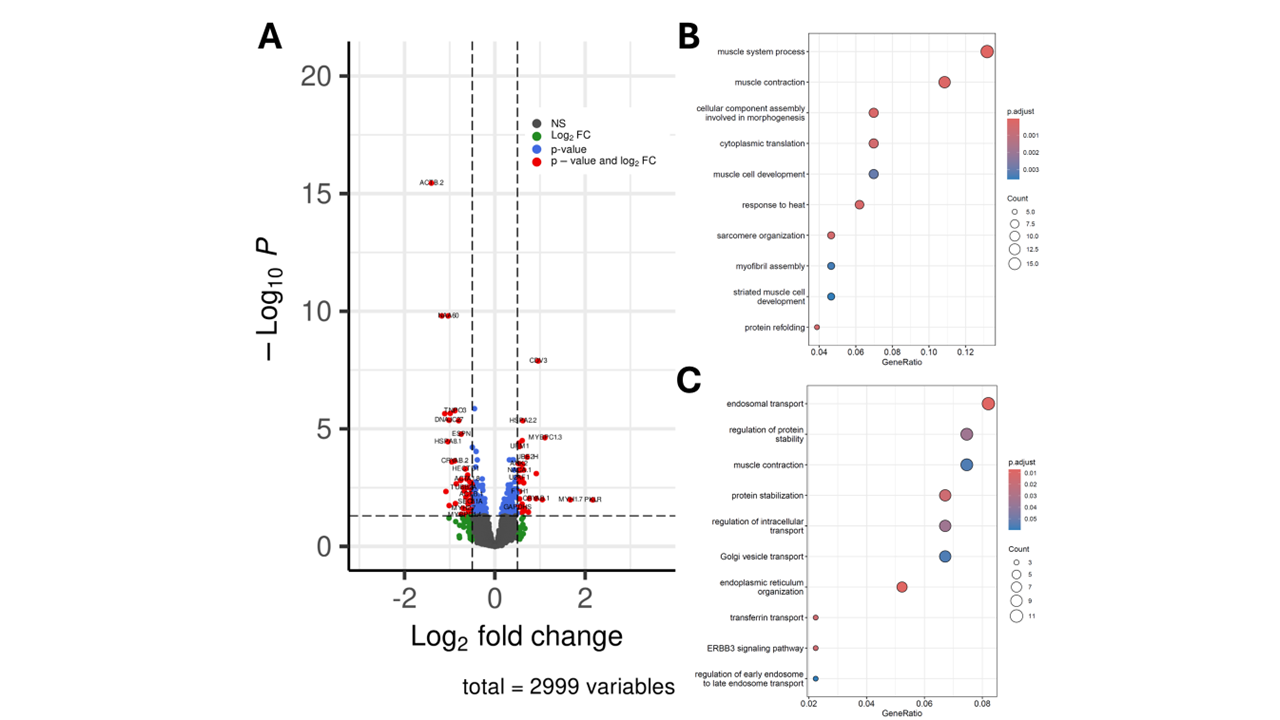

Supplement: Supplementary file 3 — FIGURE S3 Volcano plot and gene ontology biological processes changes between HDT8 and HDT1 in the control group. Note: Frame A represents the volcano plot of proteins that are differentially expressed between HDT8 and HDT1 in the control group. In the volcano plots, proteins shown in red are significantly differentially expressed, meeting both the P‐value and fold‐change criteria. Proteins in blue meet only the P‐value threshold, whereas those in green meet only the fold‐change threshold. Frame B represents the biological processes associated with upregulated proteins, and Frame C represents the biological processes associated with downregulated proteins. [file EPH-9999-0-s004.tif]

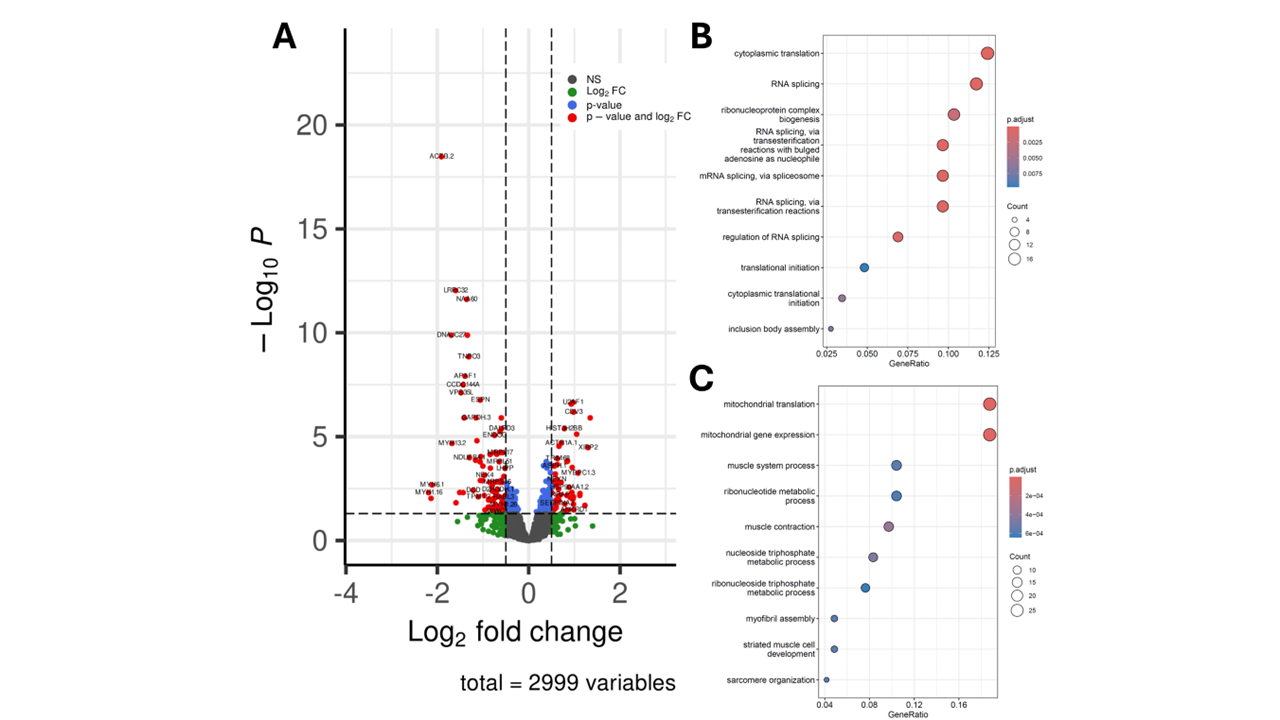

Supplement: Supplementary file 4 — FIGURE S4 Volcano plot and gene ontology biological processes changes between R7 and HDT1 in the control group. Note: Frame A represents the volcano plot of proteins that are differentially expressed between R7 and HDT1 in the control group. In the volcano plots, proteins shown in red are significantly differentially expressed, meeting both the P‐value and fold‐change criteria. Proteins in blue meet only the P‐value threshold, whereas those in green meet only the fold‐change threshold. Frame B represents the biological processes associated with upregulated proteins, and Frame C represents the biological processes associated with downregulated proteins. [file EPH-9999-0-s009.tif]

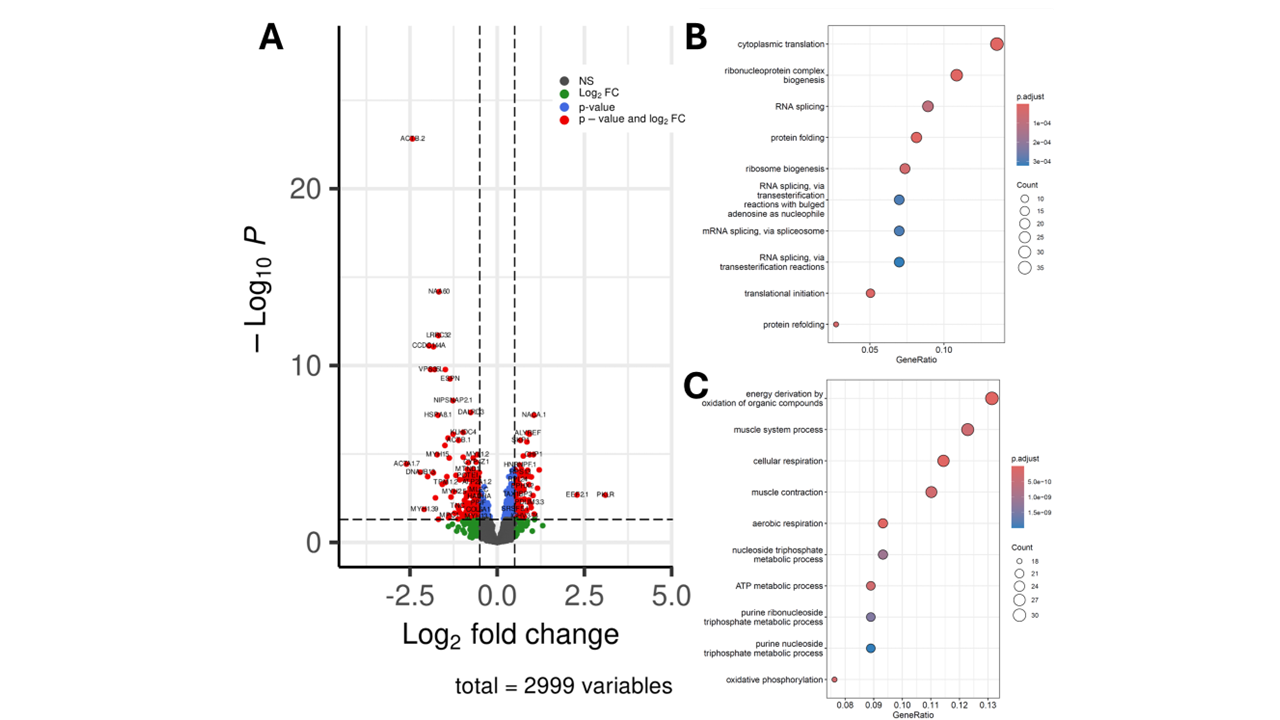

Supplement: Supplementary file 5 — FIGURE S5 Volcano plot and gene ontology biological processes changes between R35 and HDT1 in the control group. Note: Frame A represents the volcano plot of proteins that are differentially expressed between R35 and HDT1 in the control group. In the volcano plots, proteins shown in red are significantly differentially expressed, meeting both the P‐value and fold‐change criteria. Proteins in blue meet only the P‐value threshold, whereas those in green meet only the fold‐change threshold. Frame B represents the biological processes associated with upregulated proteins, and Frame C represents the biological processes associated with downregulated proteins. [file EPH-9999-0-s006.tif]

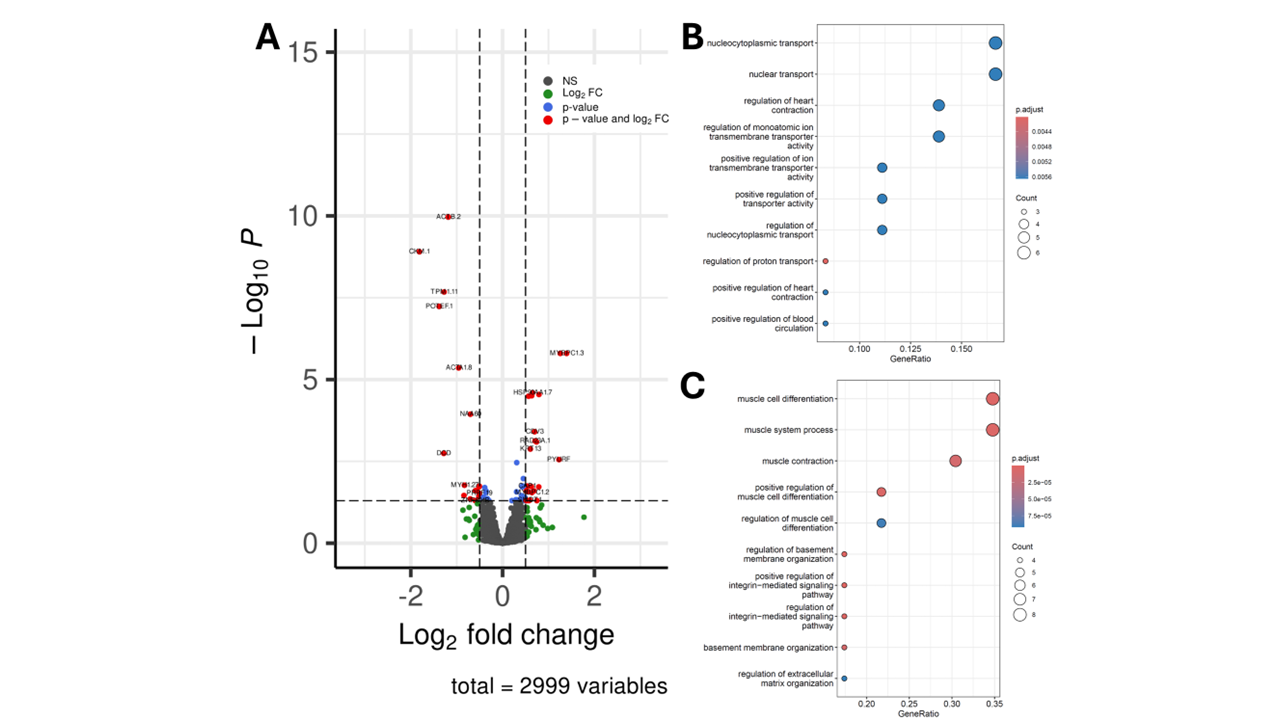

Supplement: Supplementary file 6 — FIGURE S6 Volcano plot and gene ontology biological processes changes between HDT3 and HDT1 in the exercise group. Note: Frame A represents the volcano plot of proteins that are differentially expressed between HDT3 and HDT1 in the exercise group. In the volcano plots, proteins shown in red are significantly differentially expressed, meeting both the P‐value and fold‐change criteria. Proteins in blue meet only the P‐value threshold, whereas those in green meet only the fold‐change threshold. Frame B represents the biological processes associated with upregulated proteins, and Frame C represents the biological processes associated with downregulated proteins. [file EPH-9999-0-s007.tif]

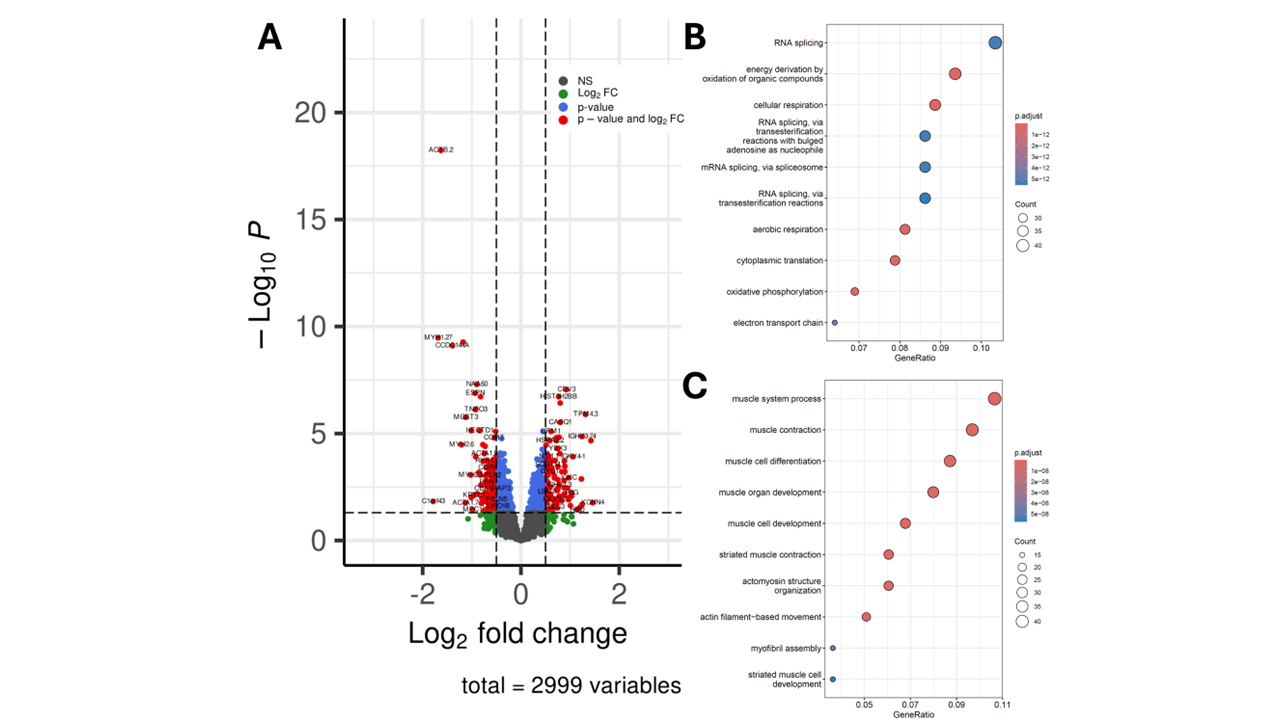

Supplement: Supplementary file 7 — FIGURE S7 Volcano plot and gene ontology biological processes changes between HDT8 and HDT1 in the exercise group. Note: Frame A represents the volcano plot of proteins that are differentially expressed between HDT8 and HDT1 in the exercise group. In the volcano plots, proteins shown in red are significantly differentially expressed, meeting both the P‐value and fold‐change criteria. Proteins in blue meet only the P‐value threshold, whereas those in green meet only the fold‐change threshold. Frame B represents the biological processes associated with upregulated proteins, and Frame C represents the biological processes associated with downregulated proteins. [file EPH-9999-0-s008.tif]

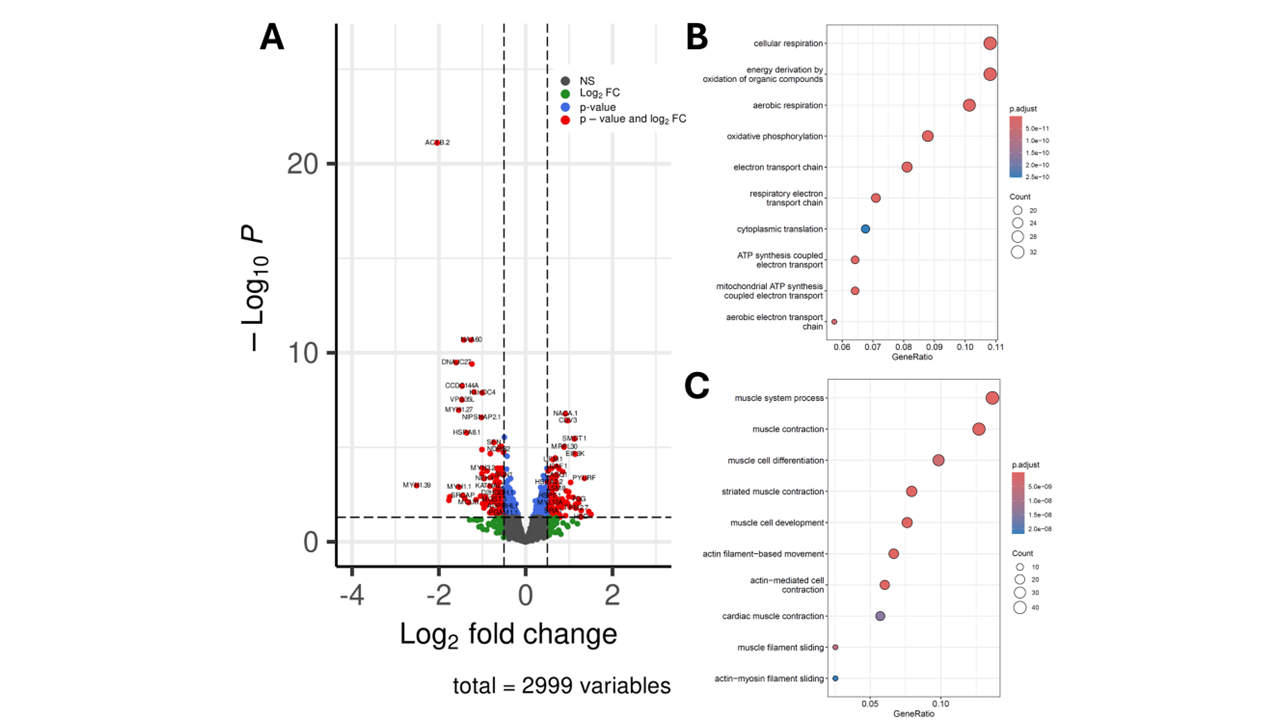

Supplement: Supplementary file 8 — FIGURE S8 Volcano plot and gene ontology biological processes changes between R7 and HDT1 in the exercise group. Note: Frame A represents the volcano plot of proteins that are differentially expressed between R7 and HDT1 in the exercise group. In the volcano plots, proteins shown in red are significantly differentially expressed, meeting both the P‐value and fold‐change criteria. Proteins in blue meet only the P‐value threshold, whereas those in green meet only the fold‐change threshold. Frame B represents the biological processes associated with upregulated proteins, and Frame C represents the biological processes associated with downregulated proteins. [file EPH-9999-0-s010.tif]

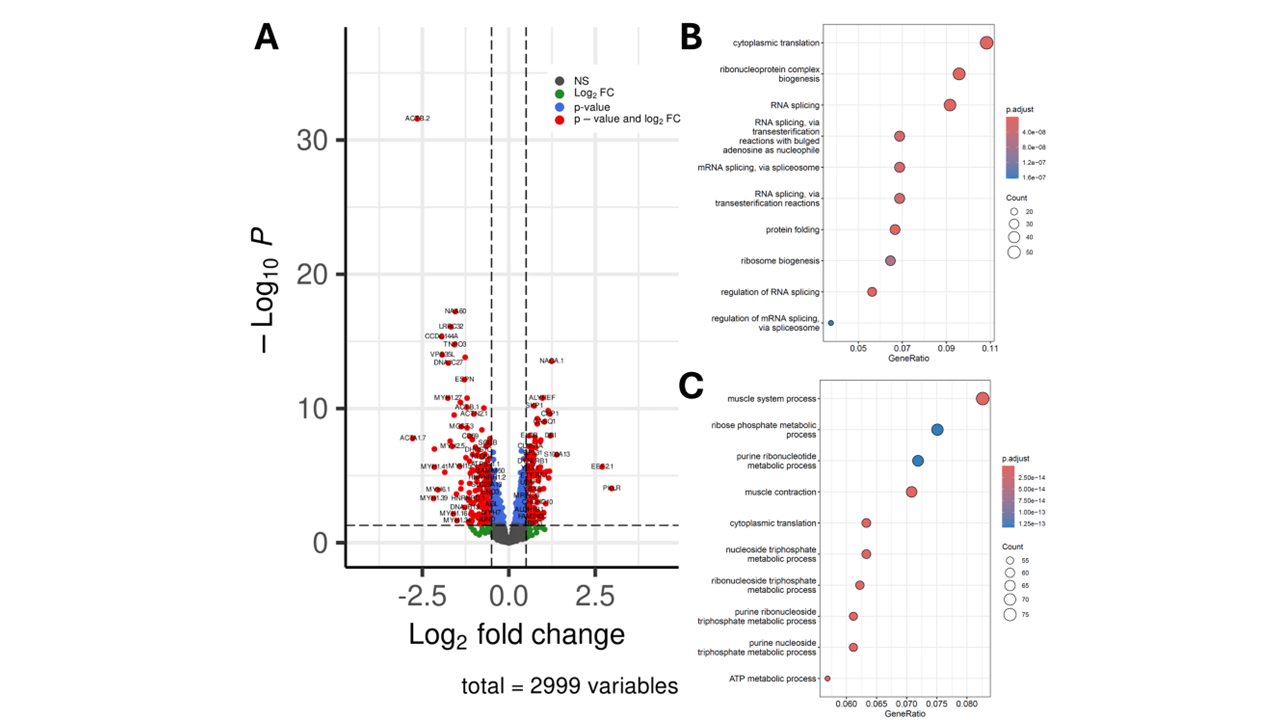

Supplement: Supplementary file 9 — FIGURE S9 Volcano plot and gene ontology biological processes changes between R35 and HDT1 in the exercise group. Note: Frame A represents the volcano plot of proteins that are differentially expressed between R35 and HDT1 in the exercise group. In the volcano plots, proteins shown in red are significantly differentially expressed, meeting both the P‐value and fold‐change criteria. Proteins in blue meet only the P‐value threshold, whereas those in green meet only the fold‐change threshold. Frame B represents the biological processes associated with upregulated proteins, and Frame C represents the biological processes associated with downregulated proteins. [file EPH-9999-0-s001.tif]

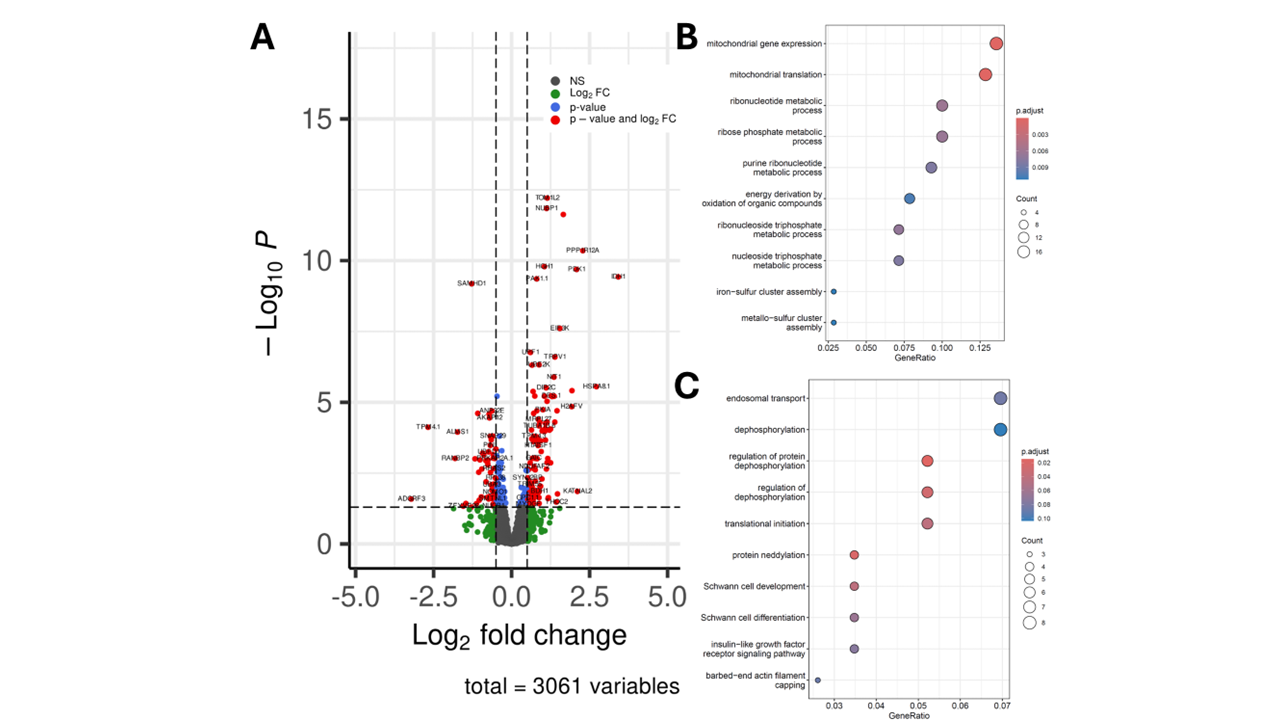

Supplement: Supplementary file 10 — FIGURE S10 Volcano plot and gene ontology biological processes differences between groups at HDT14. Note: Frame A represents the volcano plot of proteins that are differentially expressed between groups at HDT14. In the volcano plots, proteins shown in red are significantly differentially expressed, meeting both the P‐value and fold‐change criteria. Proteins in blue meet only the P‐value threshold, whereas those in green meet only the fold‐change threshold. Frame B represents the biological processes associated with upregulated proteins in the exercise compared with control group, and Frame C represents the biological processes associated with downregulated proteins in exercise compared with the control group. [file EPH-9999-0-s005.tif]
